# Efficacy of pharmacological and microbiota-based therapies in preclinical models of autism spectrum disorder: a systematic review

**DOI:** 10.1038/s41380-026-03663-8

**Published:** 2026-06-04

**Authors:** Arnas Kunevičius, Kinga Gawlińska, Aurelijus Burokas, Dawid Gawliński

**Affiliations:** 1 Department of Biological Models, Institute of Biochemistry, Life Sciences Center, Vilnius University, Sauletekio av. 7, LT-10257 Vilnius, Lithuania; 2 Department of Clinical Pharmacy, Faculty of Pharmacy, Medical College, Jagiellonian University, Medyczna 9, PL 30-688 Kraków, Poland; 3 Department of Drug Addiction Pharmacology, Maj Institute of Pharmacology Polish Academy of Sciences, Smętna 12, PL 31-343 Kraków, Poland

**Keywords:** Autism spectrum disorders, Neuroscience

## Abstract

**Background:**

Autism spectrum disorder (ASD) is a multifactorial neurodevelopmental condition in which pharmacological and microbiota-targeted interventions are emerging as promising therapeutic avenues. Animal models are the main tool to investigate etiology, molecular mechanisms and screening for pharmacological therapies. Methodological differences, outcome measure variability, incomplete reporting, biological confounders, and overgeneralization of the results made evaluating innovative pharmacological agents challenging. These limitations in the field highlight a need for systematic and standardized research to reliably assess and translate pharmacological interventions from ASD animal models to human clinical relevance.

**Subjects:**

This systematic review synthesized efficacy evidence for pharmacological and microbiota-based therapies across established ASD animal models.

**Results:**

We identified 52 recent (2010–2025) studies that reported key ASD behavioral outcomes after pharmacological or microbiota-focused treatments. Interventions were grouped into therapeutic classes - including oxytocinergic agents, E/I balance therapeutic targets, metabolic drugs, cannabinoids, purine-based interventions and emerging targets - alongside microbiota-directed strategies such as probiotics, prebiotics, and fecal microbiota transplantation. By integrating effect directions and robustness across models, we identified most potential drug candidates, evaluated the efficacy of novel strategies, and recognized critical translational gaps. The reviewed studies demonstrate that ASD-like behavioral deficits in preclinical models can be modulated through interventions targeting diverse biological systems, including neurotransmission, neuroinflammation, metabolism, and the gut-brain axis.

**Conclusions:**

These findings support the multifactorial nature of ASD pathophysiology which arises from a network of interacting systemic processes rather than a single molecular defect. It could explain the limited success of traditionally narrowly targeted interventions and suggest a paradigm shift into a more systemic approach.

**Figure:**
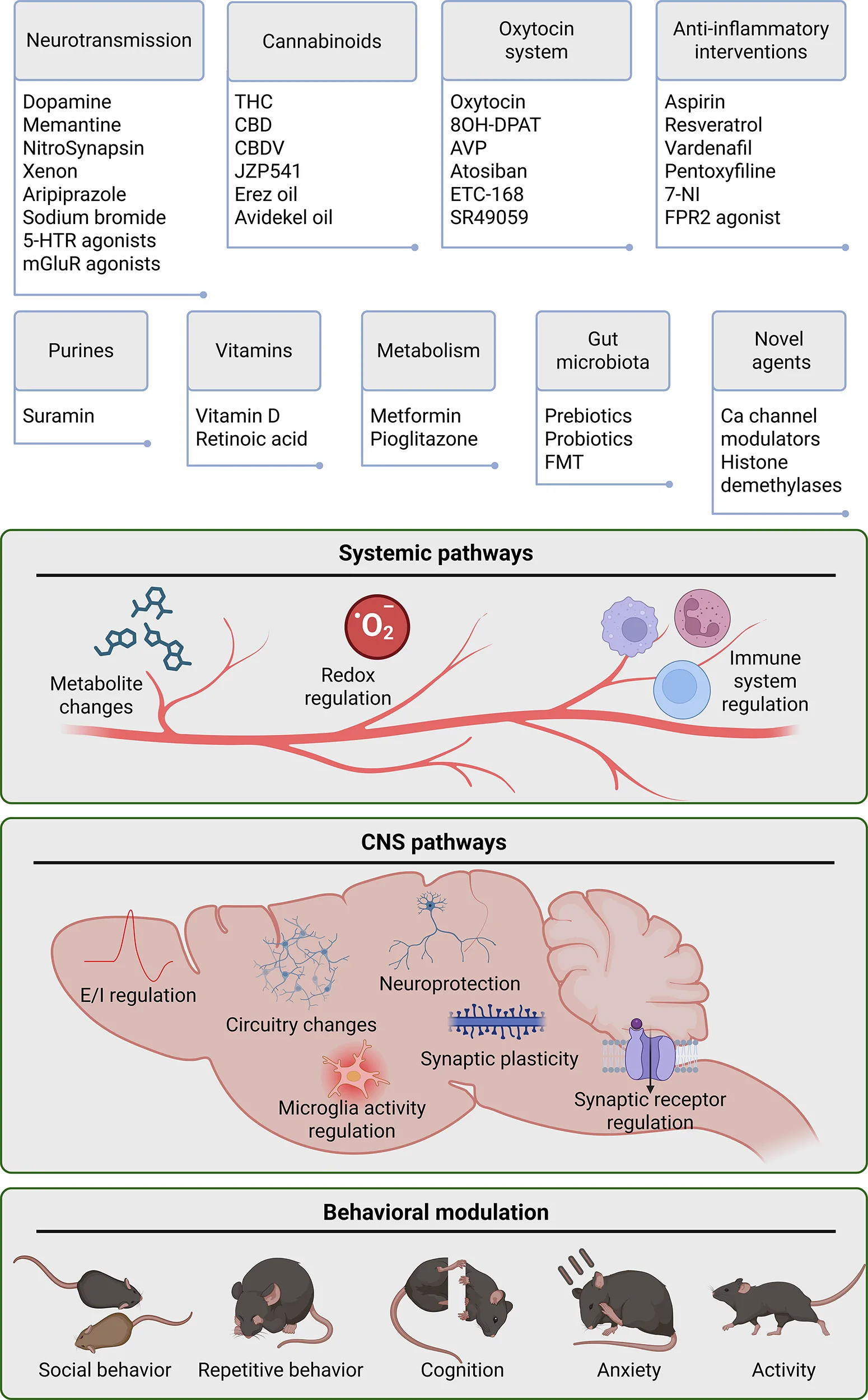

## Introduction

Autism spectrum disorder (ASD) is a neurodevelopmental disorder characterized by persistent deficits in social-emotional reciprocity, verbal and nonverbal communicative behaviors, developing and maintaining relationships as described in the fifth edition of Diagnostic and Statistical Manual of Mental Disorders, (DSM-5) (APA, [1]). ASD creates significant public health concerns both for the healthcare professionals and the patients. Lack of distinct biomarkers or clear understanding of pathogenesis makes the development of early reliable diagnostic strategies challenging [2]. Currently, behavioral interventions represent the most widely utilized clinical approach, with parent training and early intensive behavioral interventions demonstrating improvements in behavioral outcomes [3]. Although these therapies are resource-intensive and their accessibility and effectiveness can vary substantially across regions. Additionally, family factors (socio-economic situation, parental support, family structure) are important in the effectiveness of behavioral interventions [4]. These limitations in the implementation and effectiveness of behavioral interventions emphasize the need for directed pharmacological treatment options.

To date, no pharmacological intervention has been proven effective in alleviating the core symptoms of ASD. Psychopharmacological treatments are commonly employed to manage comorbid symptoms despite limited and heterogenous evidence supporting their efficacy and tolerability in individuals with ASD [5]. Medications that are typically used in ASD patients include antidepressants (selective serotonin reuptake inhibitors), stimulants (methylphenidate and amphetamine), antipsychotics (risperidone and aripiprazole), and anxiolytics (buspirone and beta-blockers) [6]. Although only risperidone and aripiprazole are approved by Food and Drug Administration (FDA) for use in individuals with ASD for irritability associated with ASD [7]. Their efficacy on core symptoms of ASD remain contested with a considerable appetite stimulation causing weight gain [8]. An analysis of psychotropic medication use among more than 30,000 children with ASD revealed that 64% were using at least one psychotropic medication, 35% were concurrently using two or more, and 15% were receiving three or more [9]. The frequent reliance on multidrug treatment strategies highlights the limited understanding of ASD pathophysiology and is based on the limited efficacy of currently available pharmacological therapies.

Animal models of ASD are the main tools for investigating etiology, molecular mechanisms and screening for pharmacologic therapies. Due to their close evolutionary relationship, rodents exhibit a high degree of genetic, biological, and neuroanatomical conservation with humans, including similar brain circuit pathways and neurotransmitter distribution, which underlie comparable behavioral phenotypes [10]. ASD rodent models exhibit multiple dimensions of validity with many robust genetic models, inbred strains mimicking the idiopathic ASD, and offering predictive value [11]. Specifically, mice and rats exhibit behavioral features that are relevant in ASD, including changes in level of activity, stereotypic, repetitive behaviors, social interactions [12].

ASD animal models can be divided into three main categories: genetic models, environmentally induced and idiopathic models [13]. The use genetic models is rooted in the substantial heritability of the ASD with at least 20% of individuals diagnosed with ASD have underlying genetic causes in de novo mutations, copy number variations, point mutations, and chromosomal changes [14]. While environmentally-induced models mostly focus on prenatal immune activation or exposure to CNS affecting drugs [15]. The development of these models were based on observations of increased risk of ASD after exposure to air pollution, pesticides, psychiatric medications and prenatal infections [16]. Although the majority of ASD cases remain idiopathic, most likely due to the interplay of multiple factors [17]. These ASD animal models are characterized by analogous behavioral deficits to those observed in patients and are useful in investigating novel genetic risk factors [18]. Currently, all these models are widely utilized in ASD research due to deficits in sociability in otherwise naturally sociable rodents.

Growing evidence indicates that immune activation and gut microbiota alterations may critically modulate both ASD pathophysiology and pharmacological treatment responses. The microbiota-gut-brain axis is a bidirectional communication system linking microbial, immune, and neural signaling pathways that regulate neurodevelopment and behavior [19, 20]. Dysregulated gut-brain-immune signaling can influence neurotransmission, neuroinflammation, and blood-brain barrier integrity, thereby altering drug metabolism, central bioavailability, and behavioral outcomes [21]. Gastrointestinal symptoms and distinct microbial profiles have been consistently reported in ASD, suggesting potential links between microbial metabolites and behavioral outcomes [22]. In the BTBR mouse model of ASD, microbiota-related changes in bile acid and tryptophan metabolism were associated with gastrointestinal dysfunction and impaired sociability [20]. Moreover, fecal microbiota transfer from ASD donors to germ-free mice induces social deficits, while probiotic and prebiotic interventions ameliorate ASD-like behaviors [23–25]. These findings highlight microbiota-targeted modulation as a promising complementary approach to pharmacological strategies in ASD.

ASD animal models have been instrumental in evaluating the effects of hundreds of pharmacological interventions, proving their value in the field [26]. However, many studies focus on general neurological symptoms rather than targeting key ASD symptoms. Additionally, methodological differences, small sample size, outcome measure variability, incomplete reporting, biological confounders, and overgeneralization of the results make evaluating innovative pharmacological agents challenging [27]. These limitations highlight a need for systematic and standardized research to reliably assess and translate pharmacological interventions from ASD animal models to human clinical relevance. To address these gaps, the objective of this systematic review is to evaluate the efficacy of currently investigated pharmacological and microbiota-targeted interventions in preclinical models of ASD [28]. Understanding how pharmacological and microbiota-targeted interventions converge on shared signaling pathways may therefore be critical for identifying biomarkers of treatment response and developing more effective, mechanism-based therapeutic strategies.

## Materials and methods

This systematic review was designed and reported in accordance with the Preferred Reporting Items for Systematic Reviews and Meta-Analyses (PRISMA) guidelines [29]. Detailed protocol for determining the research question, literature search, selection criteria, quality assessment, data extraction, and analyzing the results can be found in Supplementary material 1.

### Eligibility criteria

We included preclinical studies conducted in rodent models (mice or rats) of ASD, encompassing genetic models, environmentally induced models, and idiopathic strains exhibiting ASD-like behavioral phenotypes. Eligible studies evaluated pharmacological or microbiota-based interventions aimed at ameliorate ASD-relevant outcomes, compared to vehicle, untreated ASD controls, or wild-type/sham controls. Primary outcomes included core ASD-relevant behavioral domains (social interaction, vocalizations, restricted/repetitive behaviors). Secondary outcomes comprised molecular, neurochemical, immune, metabolic, or microbiota-related markers linked to ASD pathophysiology. Studies were eligible regardless of administration route, dosing regimen, treatment duration, or sex, provided that interventions were applied postnatally to maintain translational relevance.

Exclusion criteria included non-rodent species, animal models centered on general neurological or psychiatric conditions not specific to ASD, and non-pharmacological or non-gut microbiota interventions (behavioral, dietary, device, surgical, herbal only). Studies without an appropriate control group, studies with small sample sizes (n < 4 per group), studies lacking ASD-specific behavioral outcomes, and studies reporting insufficient methodological information were also excluded.

### Search strategy

We performed a detailed systematic search PubMed, Web of Science, ScienceDirect databases. These databases were chosen due to their extensive coverage of biomedical literature. The search was conducted between June and July of 2025 and included original research articles from January 1^st^, 2010 to June 1^st^, 2025. Key concepts were derived using the PICO framework and included keywords relevant to ASD, rodents, and intervention options. Appropriate database filters were applied to restrict results according to the publication date, study type, species, and English language. Full protocol with comprehensive search strategy, filters, keywords, and combinations is available in Supplementary material 1.

### Screening process

All records retrieved from the database searches were first subjected to automated and manual deduplication and removal of non-original research. Two reviewers (A.K., D.G.) independently screened the titles and abstracts of all remaining studies independently, using a previously prepared selection protocol (Supplementary material 1). Discrepancies between the two reviewers were resolved with a consultation from a third reviewer (A.B.). Selected publications were full-text screened independently by 3 researchers (A.K., D.G., K.G.) to assess the suitability to eligibility criteria. Reasons for exclusions were recorded (Supplementary material 2).

### Data extraction

Data extraction was conducted in duplicates and independently, using a standardized form to capture animal model details (species, strain, ASD model, sex, age), intervention type (dosage, route, frequency, duration), outcome measures (behavioral assessments, gene expression, neurotransmitters, immune signaling, hormonal measures, gut microbiota composition), and study limitations or any observed adverse effects on the animals.

### Risk of bias assessment

The methodological quality and risk of bias of included studies were evaluated by two researchers (D.G., A.K.) using the SYRCLE’s Risk of Bias tool [30]. This tool, adapted from the Cochrane Collaboration’s RoB framework for use in preclinical animal research, allows systematic evaluation of potential sources of bias across ten domains grouped into five main categories: selection, performance, detection, attrition, and reporting bias. Each domain was rated as presenting a low, high, or unclear risk of bias, according to predefined criteria.

## Results

**Fig. 1:**
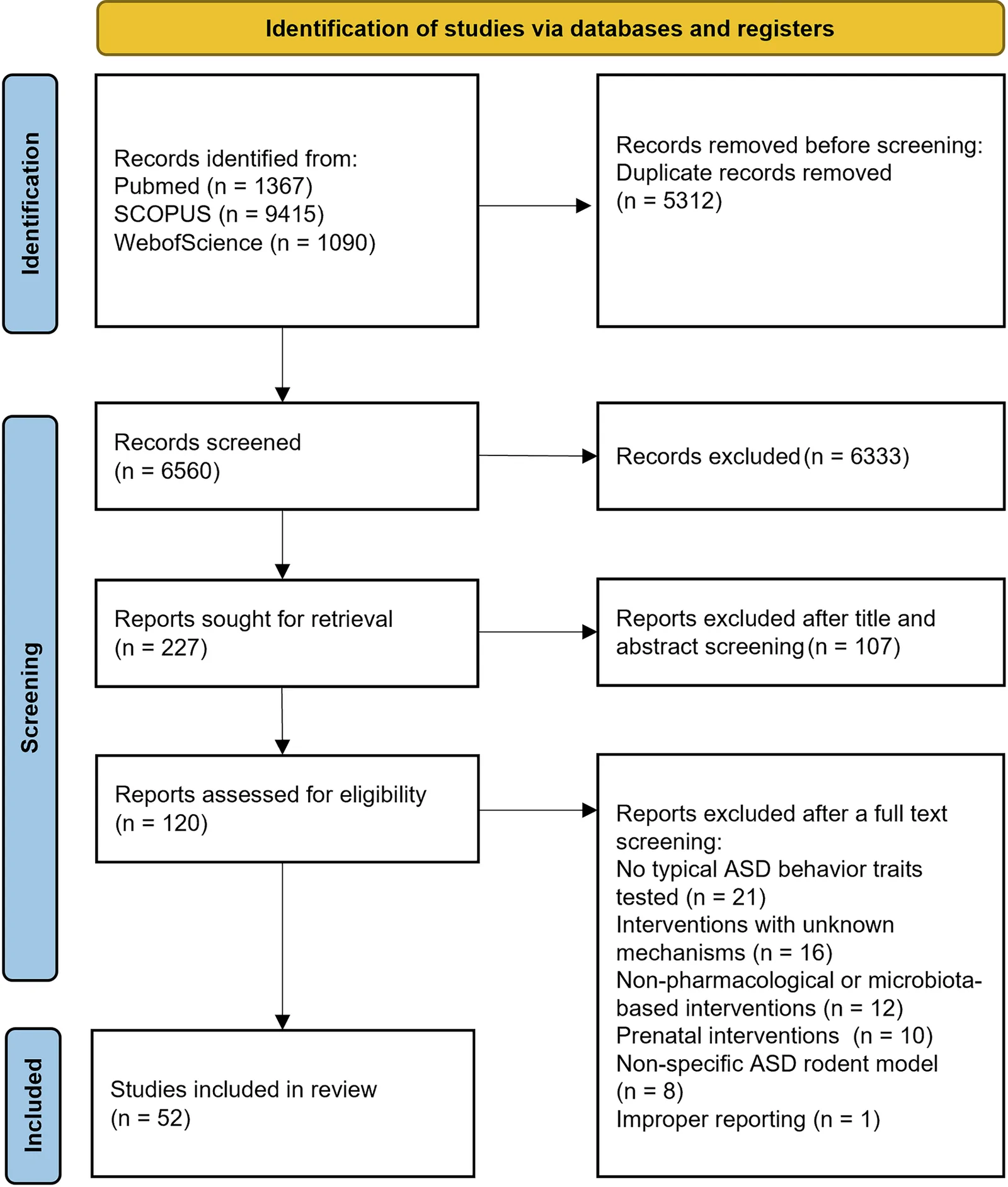
PRISMA flow diagram showing study selection from PubMed, Web of Science, ScienceDirect databases.

A total of 11872 articles were identified across the three electronic databases (PubMed, Web of Science, ScienceDirect). Automation tools were used and 5312 studies were removed as duplicates or non-original research articles. According to the PICO structure (Supplementary material 1), automated screening selected 227 studies for further title and abstract review. Next round of screening selected 120 articles for full text screening and their conformation with the inclusion/exclusion criteria. After full text evaluation, and 52 studies met criteria and were included in the systematic review. PRISMA flowchart outlined our selection process (Fig. 1). The included articles were published between 2011 and 2025 with 72% published in the past five years. This exemplifies the relevance of investigating pharmacological ASD treatment strategies.

**Fig. 2:**
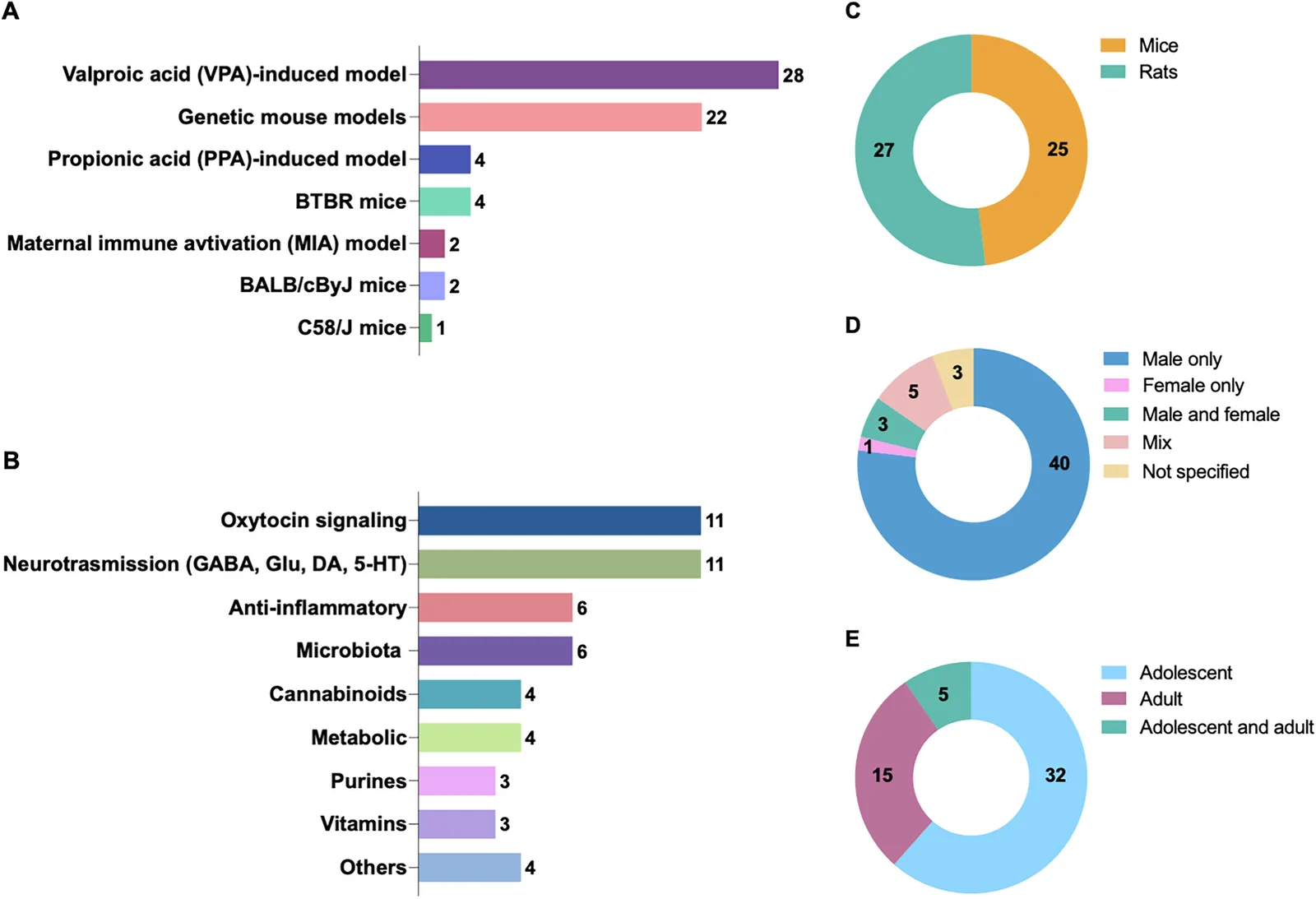
Summary of basic information from the analyzed publications. **A** Number of ASD animal models used in the analyzed studies – in some cases, more than one model was applied; (**B**) Number of publications categorized according to the therapeutic target of the interventions; (**C**) Number of publications employing mouse or rat models; (**D**) Number of studies stratified by the sex of rodents used (Mix – study where both males and females were included in the same test group (mixed-gender group); (**E**) Number of studies stratified by the age of the animals used for behavioral tests.

The systematic review contained studies that utilized 18 different ASD rodent models with the most prevalent (28 studies) being prenatal VPA exposure models (Fig. 2A). Analyzed interventions can roughly be separated into nine categories with oxytocin being the most frequently researched intervention (Fig. 2B). Of all included studies, 25 were conducted in mice and 27 in rats (Fig. 2C). The majority focused exclusively on male rodents (Fig. 2D). Our inclusion criteria ensured that all studies contained ASD-like symptom specific behavioral testing. To ensure higher translational relevance, only studies implementing postnatally administered interventions were selected, most of which (32) investigated effects in adolescent animals (Fig. 2E).

### Oxytocin signaling in preclinical ASD-like behaviors treatment

Oxytocin is a neuropeptide produced in the hypothalamus and secreted by the pituitary gland. Oxytocin-containing neural projections, together with their G protein-coupled type receptors (OXTR), are distributed across central nervous system regions that form a network known as the “social brain” [31, 32]. As a neurohormone, oxytocin exerts potent regulatory effects on social behavior, cognition, affiliation, and reward [33, 34]. Due to its functional profile, oxytocin was proposed as a potential contributor to the ASD pathophysiology as early as the late 20th century, [35, 36], prompting interest in therapies targeting oxytocinergic signaling for the core ASD symptoms. Among the studies included in this systematic review, ten directly and one indirectly evaluated the effects of pharmacological interventions modulating this signaling pathway.

It is worth noting that the preclinical ASD models used in these studies show baseline dysregulation of the oxytocin system. For example, the VPA-induced rat model is characterized by reduced oxytocin mRNA levels, fewer oxytocin-immunoreactive cell numbers in the paraventricular nucleus, and lower oxytocin concentration in the cerebrospinal fluid [31]. Similarly, genetic models such as *Cntnap2*-knockout (KO), *Oprm1*-KO, and BTBR mice also demonstrate dysregulated central oxytocin levels [31]. In turn, mutations in genes encoding OXT or its receptor (e.g., *Oxt*-KO or *Oxtr-*KO mice) result in deficits in social recognition and reward-related signaling [33, 37].

The most commonly evaluated interventions included the administration of exogenous oxytocin delivered either intranasally or via peripheral routes (intraperitoneal, subcutaneous) [31, 34, 38–41]. The efficacy of oxytocin in mitigating ASD-like behaviors depended on the treatment regimen, the developmental stage, and the sex of the animals, as well as the specific ASD model employed (Table 1). When administered acutely, intranasal oxytocin increased sociability in *Oprm1*-KO and C58/J mice [38, 42] as well as in a rat model of prenatal VPA exposure [41], but not in BALB/cByJ mice [38]. Similar outcomes were reported after a single intracerebral oxytocin administration in *Oxtr*-KO mice [33].

**Table 1:** Summary of behavioral outcomes after pharmacological and microbiota-based interventions in rodent models of ASD.

Reference	Animal model	Intervention	Social behaviors	Repetitive behaviors	Anxiety-like and exploratory behaviors	Cognitive and other behaviors
***Oxytocin signaling***						
**Bao et al**., [44]	Prenatal valproic acid (VPA)-induced, Wistar rats	Arginine vasopressin (*intranasal*, 400 µg/kg, 22 d)	**3‑chamber test:** ↑ time in social or social novelty chamber - ↑ sociability and social novelty preference	Not assessed	Not assessed	Not assessed
**Dai et al**., [31]	Prenatal valproic acid (VPA)-induced, Wistar rats	Oxytocin (*intranasal*, 20 µg; s.c., 3 µg, 7 d)	**3‑chamber test:** ↑ time in social chamber and social sniffing - ↑ sociability *(intranasal and s.c.)* **Isolation-induced pup ultrasonic vocalization:** tendency to ↑ number of ultrasonic vocalizations *(s.c.)*	**Self‑grooming test:** ↓ self-grooming time *(s.c.)*	**Light-dark box test:** tendency to ↑ time in light sight *(s.c.)*	Not assessed
**Hörnberg et al**., [37]	*Nlgn3*-KO mice; *Fmr1*-KO mice	ETC-168 (*p.o*., 5 mg/kg, acute or 8-11 d); L-368,899 (oxytocin receptor antagonist, *i.p*., 10 mg/kg)	*Nlgn3*-KO mice **Social recognition test:** ↑ time of social interaction and social recognition index *(ETC-168, 8 d)* ↓ time of social interaction and social recognition index *(L-368,899)*	*Nlgn3*-KO mice **Marble burying test:** ↔ number of buried marbles *(ETC-168,11 d)*	*Nlgn3*-KO mice **Open field test:** ↔ time in center and velocity *(ETC-168,10 d)*	*Nlgn3*-KO mice **Novel object recognition test:** ↔ object recognition index *(ETC-168, 9 d)*
						*Fmr1*-KO mice **Place-independent cue discrimination and reversal task:** ↑ number of consecutive correct responses *(ETC-168, 8 d)*
**Lindenmaier et al**., [40]	*16p11.2*^+/−^ mice; *Fmr1*-KO mice; *Shank3*-KO mice	Oxytocin (*intranasal*, 0.15 μg/10 μL, 28 d)	*16p11.2*^+/−^ mice **3‑chamber test:** ↔ time in social chamber	*16p11.2*^+/−^ mice **Self‑grooming test:** ↔ time and number of self-grooming	*16p11.2*^+/−^ mice **Open field test:** ↑ time in center, ↔ distance travelled **Rotarod test:** ↔ latency to fall	*16p11.2*^+/−^ mice **Rotarod test:** ↔ number of pre-trials to learn rotarod
			*Fmr1*-KO mice **3‑chamber test:** ↔ time in social chamber	*Fmr1*-KO mice **Self‑grooming test:** ↑ number of self-grooming	*Fmr1*-KO mice **Open field test:** ↔ time in center, distance travelled **Rotarod test:** ↔ latency to fall	*Fmr1*-KO mice **Rotarod test:** ↔ number of pre-trials to learn rotarod
			*Shank3*-KO mice **3‑chamber test:** ↔ time in social chamber	*Shank3*-KO mice **Self‑grooming test:** ↔ time and number of self-grooming	*Shank3*-KO mice **Open field test:** ↔ time in center, distance travelled **Rotarod test:** ↔ latency to fall	*Shank3*-KO mice **Rotarod test:** ↔ number of pre-trials to learn rotarod
**Liu et al**., [43]	Prenatal valproic acid (VPA)-induced, Sprague Dawley rats	Atosiban (oxytocin receptor antagonist, *intranasal*, 100 μg/kg, 14 d)	**3‑chamber test:** ↑ time in social or social novelty chamber - ↑ sociability and social novelty preference **The olfactory habituation/dishabituation test:** ↑ time smelling the social odor	**Marble burying test:** ↓ number of buried marbles	**Elevated plus maze test:** ↑ time in open arm **Open field test:** ↑ number of central zone entries and locomotor activity	Not assessed
**Moy et al**., **[**39**]**	BALB/cByJ mice	Oxytocin (*i.p*., 1 or 2 mg/kg, acute or 4 d); TC-OT-39 (oxytocin analog, *i.p*., 30 or 50 mg/kg, acute or 4 d); [pGlu4,Cyt6]OT(4–9) (*i.p*., 1 or 2 mg/kg); [Cyt6] OT(5–9) (*i.p*., 1 or 2 mg/kg); Carbetocin (*i.p*., 3, 6, 10, 15, 20 mg/kg)	**3‑chamber test:** ↑ time in social chamber - ↑ sociability *(oxytocin)* ↔ time in social chamber and social sniffing *(TC-OT-39)* ↔ time in social chamber (*OT(4–9) 0.5 and 1 mg/kg, OT(5–9) 1 or 2 mg/kg, carbetocin 20 mg/kg)* ↑ time in social chamber - ↑ sociability *(OT(4–9) 2 mg/kg)*	**Marble burying test:** ↓ number of buried marbles *(oxytocin, TC-OT-39)* ↔ number of buried marbles *(OT(4–9), OT(5–9), carbetocin)*	**Open field test:** ↓ time in center, rearing movements and distance travelled *(oxytocin, OT(4–9))*	Not assessed
**Nagano et al**., [34]	*15q dup* mice	Oxytocin (*s.c*., 0.2-0.26 mg/kg, 15 d); 8OH-DPAT (5-HT_1A_ receptor agonist; *s.c*., 0.5 mg/kg, 15 d)	**3‑chamber test:** ↑ time in social chamber - ↑ sociability *(oxytocin, 8OH-DPAT)*	Not assessed	**Open field test:** ↔ time in center and distance travelled *(oxytocin, 8OH-DPAT)*	Not assessed
**Pantouli et al**., [42]	*Oprm1-*KO mice	Oxytocin (*intranasal*, 0.15 IU ∼400 μg/kg, 0.3 IU ∼800 μg/kg or 0.6 IU ∼1600 μg/kg, acute or 17 d); LI183 (oxytocin receptor antagonist, *i.p*., 7.5 or 15 mg/kg)	**Reciprocal social interaction test:** ↑ time and number of nose contacts, paw contacts, number of followings; ↓ grooming after social contact *(oxytocin 0.3 IU acute and chronic)* ↔ time of nose contacts, paw contacts, grooming after social contact *(LI183 acute)* **3‑chamber test:** ↑ social preference *(oxytocin 0.3 IU acute and chronic)*, ↔ social novelty preference *(oxytocin 0.3 IU acute)* *More parameters in publication’s supplementary materials	**Marble burying test:** ↔ number of buried marbles *(oxytocin acute and chronic)* **Motor stereotypies test:** ↔ number of self-grooming, circling and head shakes *(oxytocin acute and chronic)*	**Novelty-suppressed feeding:** ↓ latency to feed *(oxytocin 0.6 IU acute)*; ↔ latency to feed *(oxytocin 0.3 IU chronic)*	**Y-maze test:** ↔ % of perseverative behaviors and number of arm entries *(oxytocin acute and chronic)* **Tail‑immersion nociceptive test:** ↑ time of flicking latency *(oxytocin acute and chronic)*
**Sala et al**., [33]	*Oxtr*-KO mice	Oxytocin (*i.c.v*., 0.5 ng); Vasopressin (*i.c.v*., 0.5 ng); SR49059 (V1a receptor antagonist, (*i.c.v*., 0.5 ng)	**3‑chamber test:** ↑ time in social or social novelty chamber - ↑ sociability and social novelty preference *(oxytocin and vasopressin)* ↔ time in social or social novelty chamber *(SR49059)*	Not assessed	**Aggression test:** ↓ number of attacks and tail-rattling, ↑ attack latency *(oxytocin and vasopressin)*, ↔ number of attacksand tail-rattling, attack latency *(SR49059)* **Locomotor activity:** ↔ number of horizontal counts *(oxytocin, vasopressin and SR49059)*	**T-maze test:** ↓ number of days to reach criterion *(oxytocin and vasopressin)*, ↔ number of days to reach criterion *(SR49059)*
**Shariatpanahi et al**., [41]	Prenatal valproic acid (VPA)-induced, Wistar rats	Oxytocin (*intranasal*, 1 μg/μL, 10 μL per nostril)	**3‑chamber test:** ↑ time in social or social novelty chamber in male and female - ↑ sociability and social novelty preference	**Self‑grooming test:** ↓ self-grooming time in male and female **Marble burying test:** ↓ number of buried marbles in male and female	Not assessed	**Morris water maze test:** ↓ escape latency in male and female, ↔ velocity in male and female
**Teng et al**., [38]	BALB/cByJ mice; C58/J mice	Oxytocin (*i.p*., 1 mg/kg, acute or 4 d)	C58/J mice **3‑chamber test:** ↑ time in social chamber - ↑ sociability after 2 weeks in male and after 1 or 2 weeks in female	C58/J mice **Self‑grooming test:** ↑ self-grooming time and ↓ locomotion time *(acute oxytocin)*, ↔ self-grooming and locomotion time *(4 d oxytocin*)	C58/J mice **Open field test:** ↔ time in center and distance travelled	C58/J mice Not assessed
			BALB/cByJ mice **3‑chamber test:** ↔ time in and entries into social chamber *(acute oxytocin)*; ↑ time in social chamber and social sniffing time - ↑ sociability *(4 d oxytocin*)	BALB/cByJ mice Not assessed	BALB/cByJ mice Not assessed	BALB/cByJ mice Not assessed
***Neurotrasmission (GABA, glutamete, DA, 5-HT)***						
**Chao et al**., [60]	BTBR mice; *Fmr1*-KO mice	Dopamine hydrochloride (*intranasal*, 3 mg/kg)	*BTBR mice* **3‑chamber test:** ↑ time in social chamber - ↑ sociability	*BTBR mice* **Open field test:** ↔ self-grooming time	*BTBR mice* **Open field test:** ↔ distance travelled and time in center, ↓ thigmotactic behaviors, ↑ rearing time in the first 5 min **Elevated plus maze test:** ↔ distance travelled, entries to and time spent in the center, open and closed arms, and counts of head-dips	*BTBR mice* **Object‑based attention test:** ↑ time with novel object
			*Fmr1-KO mice* **3‑chamber test:** ↑ time in social or social novelty chamber - ↑ sociability and social novelty preference	*Fmr1*-*KO mice* **Open field test:** ↔ self-grooming time	*Fmr1*-*KO mice* **Open field test:** ↔ distance travelled, time in center, thigmotactic behaviors and rearing time **Elevated plus maze test:** ↔ distance travelled, entries to and time spent in the center, open and closed arms, and counts of head-dips	*Fmr1-KO mice* **Object‑based attention test:** ↑ time with novel object
**D’Addario et al**., [57]	*Fmr1*-KO mice	PD158780 (ErbB inhibitor, *i.c.v*. into SNpc, 10 µM or *i.p*., 10 mg/kg)	Not assessed	**Self‑grooming test:** ↓ self-grooming time **Marble burying test:** ↓ number of buried marbles	**Open field test:** ↔ distance travelled	Not assessed
**Derieux et al**., [55]	*Oprm1-*KO mice*;*	NaBr (*i.p*., 10, 30, 70, 125, 250 or 500 mg/kg, 18 d); NaBr (*i.p*., 250 mg/kg, 15 d); NaBr (*p.o*., 250 mg/kg, 5 d); KBr (*i.p*., 145 mg/kg, 18 d) Bumetanide (*i.p*., 0.5, 2 mg/kg, 18 d); VU0155041 (PAM of mGlu4 receptor, *i.p*., 1 mg/kg, 18 d); NaBr (*i.p*., 70, mg/kg, 18 d) + VU0155041 (*i.p*., 1 mg/kg, 18 d)	*Oprm1-*KO mice **Reciprocal social interaction test:** ↑ number and time of nose contacts *(NaBr 125-500 mg/kg, NaBr 15 d*, *KBr, NaBr 250 mg/kg p.o, NaBr 70 mg/kg* + *VU0155041 1 mg/kg);* ↓ grooming after social contact *(NaBr 70-500 mg/kg, NaBr 15 d, KBr, NaBr 250 mg/kg p.o., bumetanide);* ↔ number and time of nose contacts *(bumetanide, VU015504*1*)* **3‑chamber test:** ↑ time in social sniffing - ↑ sociability *(NaBr 30-500 mg/kg, KBr, NaBr 70 mg/kg* + *VU0155041 1 mg/kg);* ↔ time in social sniffing *(bumetanide, VU015504*1*)*	*Oprm1-*KO mice **Motor stereotypies test:** ↓ number of circling and head shakes *(NaBr 125-500 mg/kg, KBr, NaBr 250 mg/kg p.o., bumetanide)*, ↓ number head shakes *(NaBr 70 mg/kg* + *VU0155041 1 mg/kg)*	*Oprm1-*KO mice **Novelty-suppressed feeding test:** ↓ latency to feed *(NaBr 10-500 mg/kg, KBr, NaBr 70 mg/kg* + *VU0155041 1 mg/kg);* ↔ latency to feed *(bumetanide, VU0155041 1 mg/kg)*	*Oprm1-*KO mice **Y‑maze test:** ↓ perseverative errors *(NaBr 30-500 mg/kg, Bumetanide 0.5 mg/kg, VU0155041 1 mg/kg, NaBr 70 mg/kg* + *VU0155041 1 mg/kg)*
	*Fmr1*-KO mice;		*Fmr1-KO* mice **Reciprocal social interaction test:** ↑ number and time of nose contacts; ↓ grooming after social contact *(NaBr 250 mg/kg)* **3‑chamber test:** ↑ time in social sniffing - ↑ sociability *(NaBr 250 mg/kg)*	*Fmr1-KO* mic*e* **Motor stereotypies test:** ↓ number of circling and head shakes *(NaBr 250 mg/kg)*	*Fmr1-KO* mice **Novelty-suppressed feeding test:** ↓ latency to feed *(NaBr 250 mg/kg)*	*Fmr1-KO* mice **Y‑maze test:** ↓ perseverative errors *(NaBr 250 mg/kg)*
	*Shank3*^Δex13-16−/−^ mice		*Shank3*^*Δex13-16−/−*^ mice **Reciprocal social interaction test:** ↑ number and time of nose contacts; ↓ grooming after social contact *(NaBr 250 mg/kg)* **3‑chamber test:** ↑ time in social sniffing - ↑ sociability *(NaBr 250 mg/kg)* *More parameters in publication’s supplementary materials	*Shank3*^*Δex13-16−/−*^ mice **Motor stereotypies test:** ↓ number of circling and head shakes *(NaBr 250 mg/kg)*	*Shank3*^*Δex13-16−/−*^ mice **Novelty-suppressed feeding test:** ↓ latency to feed *(NaBr 250 mg/kg)*	*Shank3*^*Δex13-16−/−*^ mice **Y‑maze test:** ↓ perseverative errors *(NaBr 250 mg/kg)*
**Dobrovolsky et al**., [50]	Valproic acid (VPA)-induced, Wistar rats (exposure pups)	Xenon (25% inhalation, 10 min)	**Test for social novelty:** ↓ time to leave the starting area, ↔ time spent with dam and with unfamiliar adult female **Social play behavior:** ↓ number of attacks; ↑ number of sniffings	Not assessed	**Open field test:** ↔ number of rearings, sections crossed, number of center entries and grooming **Elevated plus maze test:** ↔ time in closed arms	**Forced swim test:** ↔ climbing time
**Habib et al**., [53]	Prenatal valproic acid (VPA)-induced, Wistar rats	Risperidone (*i.p*., 1 or 3 mg/kg, 28 d)	**3‑chamber test:** ↑ sociability index and social novelty preference index - ↑ sociability and social novelty preference *(1 and 3 mg/kg)*	**Marble burying test:** ↓ number of buried marbles *(1 and 3 mg/kg)* **Open field test:** ↓ self-grooming time *(1 and 3 mg/kg)*	**Open field test:** ↑ number of crossed lines, number of entries to the central zone, time spent in the central zone, and rearing time *(1 and 3 mg/kg)*	Not assessed
**Rahdar et al**., [62]	Prenatal valproic acid (VPA)-induced, Wistar rats	LP-211 (5-HT_7_ receptor agonist, *i.p*., 1 mg/kg, 10 d)	Not assessed	**Open field test:** ↓ self-grooming time; ↓ number and time of rearing	**Open field test:** ↑ distance travelled in center and open field index, ↔ time of horizontal activity and time of freezing	**Novel object recognition test:** ↑ discrimination index **Wire hanging test:** ↑ latency to fall
**Santrač et al**., [58]	Prenatal valproic acid (VPA)-induced, Wistar rats	MP-III-022 (α5GABAAR PAM, *i.p*., 0.33 or 1 mg/kg, 7 d)	**Reciprocal social interaction test:** ↑ time spent in allogrooming and allosniffing *(0.33 mg/kg in male)*, ↓ time spent in crawling over/under conspecific *(0.33 and 1 mg/kg in male)* ↑ time spent in tail manipulation of conspecific *(1 mg/kg in female)* ↔ time spent following conspecific *(0.33 and 1 mg/kg in male and female)*	**Locomotor activity:** ↓ self-grooming time *(0.33 mg/kg in male)*	**Elevated plus maze test:** ↔ entries into open arms **Locomotor activity:** ↓ active time *(0.33 mg/kg in male)*, ↔ number of rotations, ↓ number of clockwise rotations *(0.33 mg/kg in male)*; ↓ number of clockwise and anticlockwise rotations *(1 mg/kg in female)*	**Morris water maze test:** ↓ decreased latency, ↑ path efficiency *(0.33 mg/kg in male*
**Tu et al**., [52]	*Mef2c*^*+/−*^ mice	NitroSynapsin (*i.p*., 4.6 μmol/kg, twice a day for 90 d)	**3‑chamber test:** ↑ time in social chamber, number and duration of visits - ↑ sociability	**Hole board exploration test:** ↓ number of head-dips per hole	**Open field test:** ↓ time in center; ↔ total activity	**Morris water maze test:** ↑ time spent in target quadrant, ↔ swimming speed **Paw clasping test:** ↔ number of paw clasping
**Vicidomini et al**., [54]	*Shank3*^*Δ11 -/-*^ mice	CDPPB (mGlu5 receptor PAM, *i.p*., 3 mg/kg, acute or chronic)	**3‑chamber test:** ↑ sociability index and social novelty preference index - ↑ sociability and social novelty preference	**Self‑grooming test:** ↓ self-grooming time and number of episodes *(acute)*	**Spontaneous motor activity:** ↔ horizontal and vertical counts *(acute)*	**Morris water maze test:** ↔ escape latency to the target zone and the time spent in the quadrant (day 5) ↓ escape latency to the target zone and ↑ time spent in the quadrant (day 10)
**Yoshimura et al**., [56]	BTBR mice	GRN-529 (*i.p*., 3 mg/kg); 2-261 (*i.p*., 0.1, 0.3, 1, 3 mg/kg); 2-301 (*i.p*., 0.3, 1, 3 mg/kg); 2-313 (*i.p*., 1, 3 mg/kg) AVL-3288 (*i.p*., 0.3, 1, 3, 10 mg/kg); 4-327 (*i.p*., 1, 3 mg/kg)	**3‑chamber test:** ↑ social sniffing time - ↑ sociability *(GRN-529, 2-261 0.3-3 mg/kg; 2-301 1-3 mg/kg, 2-313 3 mg/kg; AVL-3288 3 mg/kg, 4-327 3 mg/kg)*	**Self‑grooming test:** ↓ self-grooming time *(GRN-529, AVL-3288 3-10 mg/kg, 4-327 1 mg/kg)*	**Open field test:** ↑ distance travelled (*GRN-529)*, ↔ distance travelled *(2-261, AVL-3288)*	Not assessed
**Zohny et al**., [51]	Prenatal valproic acid (VPA)-induced, Wistar rats	Memantine (*i.p*., 20 mg/kg, 38 d); Aripiprazole (*i.p*., 3 mg/kg, 38 d); Memantine (*i.p*., 20 mg/kg, 38 d) + Aripiprazole (*i.p*., 3 mg/kg, 38 d)	**3‑chamber test:** ↑ sociability index *(memantine, aripiprazole, memantine + aripiprazole)*, ↑ social novelty preference index *(memantine, memantine + aripiprazole)*	**Marble burying test:** ↓ number of buried marbles *(memantine, aripiprazole, memantine + aripiprazole)* **Open field test:** ↓ number of self-grooming *(memantine + aripiprazole)*	**Open field test:** ↑ time in center, ↔ number of total crossed squares *(memantine, aripiprazole, memantine + aripiprazole)*	**Morris water maze test:** ↔ latency to reach platform, ↑ time spent in target quadrant *(memantine, aripiprazole, memantine + aripiprazole)* **Attentional Set‑Shifting test:** ↓ number of trials to reach criterion *(memantine, aripiprazole, memantine + aripiprazole)* **Tail‑Immersion Nociceptive test:** ↓ tail withdrawal latency *(memantine + aripiprazole)*
***Anti-inflammatory***						
**Abdel-Haq et al**., [95]	*Shank3*^*Δ4-22*^ mice	7‑NI (nNOS inhibitor; *s.c*., PSARA gel formulation, 80 mg/kg)	**3‑chamber test:** ↑ time in social or social novelty chamber - ↑ sociability and social novelty preference	Not assessed	**Elevated plus maze test:** ↑ time in open arms **Open field test**: ↔ distance travelled	**Novel object recognition test:** ↑ time with novel object
**Cristiano et al**., [93]	BTBR mice; Prenatal valproic acid (VPA)-induced, B6 mice	MR‑39 (FPR2 agonist; *i.p*., 10 mg/kg, 8 d)	*BTBR mice* **3‑chamber test:** ↑ time in social chamber - ↑ sociability **Reciprocal social interaction test:** ↑ number of following, push-crawl, nose to nose, nose to anogenital sniffing behaviors	*BTBR mice* **Reciprocal social interaction test:** ↓ self-grooming episodes **Marble burying test:** ↔ number of buried marbles **Self‑grooming test:** ↔ self-grooming time	*BTBR mice* Not assessed	*BTBR mice* Not assessed
			*Prenatal valproic acid (VPA)-induced, B6 mice* **3‑chamber test:** ↑ time in social chamber - ↑ sociability **Reciprocal social interaction test:** ↑ number of followings, nose to nose sniffing behaviors	*Prenatal valproic acid (VPA)-induced, B6 mice* **Reciprocal social interaction test:** ↓ self-grooming episodes **Marble burying test:** ↔ number of buried marbles **Self‑grooming test:** ↔ self-grooming time	*Prenatal valproic acid (VPA)-induced, B6 mice* Not assessed	*Prenatal valproic acid (VPA)-induced, B6 mice* Not assessed
**Erdoğan et al**., [96]	Propionic acid (PPA)-induced, male Wistar rats	Pentoxifylline (*p.o*., 300 mg/kg, 15 d)	**3‑chamber test:** ↑ time in social chamber - ↑ sociability	Not assessed	**Open field test:** ↑ number of ambulation	**Passive avoidance learning test:** ↑ latency to move to the dark chamber, time spent in target quadrant
**Hidema et al**., [92]	*Oxtr*-KO mice; Prenatal valproic acid (VPA)-induced, C57BL6/J mice	Resveratrol (*i.p*., 30 mg/kg)	*Oxtr*-KO mice **3‑chamber test:** ↑ social novelty preference index - ↑ social novelty preference	*Oxtr*-KO mice Not assessed	*Oxtr*-KO mice Not assessed	*Oxtr*-KO mice Not assessed
			*Prenatal valproic acid (VPA)-induced, C57BL6/J mice* **3‑chamber test:** ↑ social novelty preference index - ↑ social novelty preference	*Prenatal valproic acid (VPA)-induced, C57BL6/J mice* Not assessed	*Prenatal valproic acid (VPA)-induced, C57BL6/J mice* Not assessed	*Prenatal valproic acid (VPA)-induced, C57BL6/J mice* Not assessed
**Özkul et al**., [94]	Propionic acid (PPA)-induced, male Wistar rats	Vardenafil (*p.o*., 3 mg/kg, 15 d)	**3‑chamber test:** ↑ time in social chamber - ↑ sociability	Not assessed	**Open field test:** ↑ number of ambulation	**Passive avoidance learning test:** ↑ passive avoidance learning latency
**Zhang et al**., [97]	Prenatal valproic acid (VPA)-induced, Sprague Dawley rats	Aspirin (*i.p*., 1 mg/kg, 30 d)	**3‑chamber test:** ↑ time in social chamber and sociability index - ↑ sociability	**Self‑grooming test:** ↓ self-grooming time and number of episodes	**Open field test:** ↑ time in center and ↓ distance travelled **Light-dark box test:** ↓ time in dark box, ↔ number of transitions	Not assessed
***Microbiota***						
**Abuaish et al**., [70]	Propionic acid (PPA)-induced, male Sprague Dawley rats	Fecal microbiota transplant (*p.o*., 1 g/kg/day, 22 d); *Bifidobacterium longum* BB536 (*p.o*., 1×10⁹ CFU/day, 22 d)	**3‑chamber test:** ↑ time in social chamber - ↑ sociability (*Bifidobacterium longum* BB536 > Fecal microbiota transplant); ↓ immobility (*Bifidobacterium longum* BB536 > Fecal microbiota transplant)	Not assessed	Not assessed	Not assessed
**Abujamel et al**., [66]	Propionic acid (PPA)-induced, male Sprague Dawley rats	Fecal microbiota transplant (*p.o*., 1 g/kg/day, 30 d); *Bifidobacterium longum* BB536 (*p.o*., 1 × 10⁹ CFU/day, 30 d)	**3‑chamber test:** ↑ time in social chamber - ↑ sociability (*Bifidobacterium longum* BB536 > Fecal microbiota transplant); ↓ immobility (*Bifidobacterium longum* BB536 > Fecal microbiota transplant)	Not assessed	Not assessed	Not assessed
**Kong et al**., [67]	Prenatal valproic acid (VPA)-induced, Wistar rats	*Bifidobacterium longum* CCFM1077 (*p.o*., 10⁹ CFU/mL); Risperidone (*p.o*., 1.6 g/100 g)	**3‑chamber test:** ↔ social index (*B. longum)*, ↑ social index *(risperidone)*	**Marble burying test:** ↓ number of buried marbles (*B. longum and risperidone)*	**Open field test:** ↔ time in center (*B*. *longum)*, ↑ time in center *(risperidone)*	**Y-maze test:** ↔ time in familiar arm (*B. longum and risperidone)*, ↑ time in novel arm (*B. longum)* **Forced swim test:** ↓ time of stopping and floating
**Mintál et al**., [68]	Prenatal valproic acid (VPA)-induced, Wistar rats	Probiotic mixture (*Lactobacillus spp., Bifidobacterium spp*; *p.o*., 14 d)	**3‑chamber test:** ↑ sociability index	**3‑chamber test:** ↔ number of rearing and grooming behavior	**3‑chamber test:** ↔ distance travelled	Not assessed
**Wang et al**., [71]	Prenatal valproic acid (VPA)-induced, C57BL6/J mice	Fecal microbiota transplantation from healthy patients (*p.o*., 21 d)	**3‑chamber test:** ↑ time in social chamber - ↑ sociability	Not assessed	**Open field test:** ↑ time in center and distance travelled **Elevated plus maze test:** ↑ time in open arms and number of entries into open arms	Not assessed
**Wang et al**., [69]	Prenatal valproic acid (VPA)-induced, C57BL6/J mice	*Lactobacillus reuteri C501* (*p.o*., 10^8^ CFU, 28 d); Inulin (*p.o*., 1% w/v in drinking water, 28 d); Synbiotic (*p.o*., *L. reuteri C501*, 10^8^ CFU + inulin, 1% w/v, 28 d)	**3‑chamber test:** ↑ sociability index and social novelty preference index - ↑ sociability and social novelty preference (*L. reuteri, inulin, synbiotic)*	**Self‑grooming test:** ↓ self-grooming time *(L. reuteri, inulin, synbiotic)*	Not assessed	Not assessed
***Cannabinoids***						
**Loomis et al**., [85]	Prenatal valproic acid (VPA)-induced, Wistar rats	JZP541 (*i.p*., acute 100 mg/kg or 10, 30, 100 mg/kg, 5 d); Risperidone (*i.p*., 0.125 mg/kg, 5 d)	**3‑chamber test:** ↑ time in social chamber *(10, 30, 100 mg/kg)*, ↔ time in social chamber *(risperidone)* **Maternal-pup vocalizations:** ↑ number of calls during pre-isolation and reunion periods, ↑ duration of calls during isolation period *(acute JZP541)* **Reciprocal social interaction test:** ↑ frequency *(30, 100 mg/kg)* and duration of social behavior *(30 mg/kg)*, ↔ frequency and duration of social behavior *(risperidone)*	**Marble burying test:** ↓ number of buried marbles *(10, 30, 100 mg/kg)*, ↔ number of buried marble *(risperidone)* **Open field test:** ↓ number of self-grooming and turns *(100 mg/kg and risperidone)*	**Open field test:** ↓ time in center in novel environment *(risperidone)*, ↓ time in center in familiar environment *(100 mg/kg and risperidone)*	**Bottle-brush test (irritability):** ↓ irritability score and number of aggressive responses in juvenile *(100 mg/kg)* and adult animals *(30 and 100 mg/kg)*, ↔ irritability score *(risperidone)*
**Pedrazzi et al**., [83]	Prenatal valproic acid (VPA)-induced, Swiss mice	Cannabidiol (*i.p*., 30 or 60 mg/kg)	**Reciprocal social interaction test:** ↑ social interaction time *(60 mg/kg)*	**Marble burying test:** ↓ number of buried marbles	**Actimeter test:** tendency to ↓ number of stereotyped-like movements *(60 mg/kg)*	**Novel object recognition test:** ↑ discrimination index *(30 and 60 mg/kg)* **Prepulse inhibition test (PPI):** ↑ % of prepulse inhibition *(30 and 60 mg/kg)*
**Poleg et al**., [82]	*InsG3680 Shank3* mice	Avidekel oil (*p.o*., 5 ml/kg, (CBD:THC ratio 20:1, 25 mg/kg CBD, 1 mg/kg THC, twice a week for 21 d); 5 ml/kg CBD oil (*p.o*., 25 mg/kg CBD twice a week for 21 d); Erez oil (*p.o*., 1 mg/kg THC, no CBD twice a week for 21 d); THC oil (*p.o*., 1 mg/kg THC twice a week for 21 d); AM-251 (CB1 receptor antagonist, *i.p*., 3 mg/kg); WIN55,212-2 (CB1 receptor agonist, *i.p*., 0.5 mg/kg)	**Social approach test:** ↓ time in social zone *(Avidekel oil, CBD)*, ↑ time in social zone *(Eraz oil, THC)*	**Self‑grooming test:** ↓ self-grooming time *(Avidekel oil; Eraz oil, WIN55,212-2)*, ↔ self-grooming time *(AM-251, CBD, THC)*	**Open field test:** ↓ distance travelled *(Avidekel oil)*, ↔ distance travelled *(Eraz oil, CBD, THC)* **Elevated plus maze test:** ↑ time in open arms *(Avidekel oil)*, ↔ distance travelled *(Eraz oil, CBD, THC)*	**Forced swim test:** ↔ swimming time *(Avidekel oil, Eraz oil)*
**Zamberletti et al**., [84]	Prenatal valproic acid (VPA)-induced, Sprague Dawley rats	Cannabidivarin (*i.p*., 0.2, 2, 20, 100 mg/kg, 23 d - symptomatic schedule); Cannabidivarin (*i.p*., 0.2, and 20 mg/kg, 12 d - preventive schedule)	**3‑chamber test:** ↑ time in social chamber - ↑ sociability *(20 and 100 mg/kg, 23 d)*, ↑ time in social novelty chamber - ↑ social novelty preference *(20 mg/kg, 23 d)*, ↑ time in social chamber - ↑ sociability *(2 and 20 mg/kg, 12 d)*, ↑ time in social novelty chamber - ↑ social novelty preference *(20 mg/kg, 12 d)*	**Open field test:** ↓ self-grooming time *(20 mg/kg, 23 d)*, ↔ self-grooming time *(2 and 20 mg/kg, 12 d)*	**Open field test:** ↓ distance travelled *(2, 20, 100 mg/kg, 23 d)*, ↓ distance travelled *(20 mg/kg, 12 d)*	**Novel object recognition test:** ↑ discrimination index *(2, 20, 100 mg/kg, 23 d)*, ↑ discrimination index *(20 mg/kg, 12 d)*
***Metabolic***						
**Elnahas et al**., [75]	Prenatal valproic acid (VPA)-induced, Wistar rats	Metformin (*i.p*., 100 mg/kg, 38 d); Risperidone (*i.p*., 1 mg/kg, 38 d); Metformin (*i.p*., 100 mg/kg, 38 d) + Risperidone (*i.p*., 1 mg/kg, 38 d)	**3‑chamber test:** ↑ sociability index and social novelty preference index *(metformin, risperidone and metformin + risperidone)*	**Marble burying test:** ↓ number of buried marbles *(metformin, risperidone and metformin + risperidone)*	**Open field test:** ↑ number of crossed squares, central zone entries and rearing, ↓ number of grooming *(metformin, risperidone and metformin + risperidone);* ↓ latency to leave central zone *(metformin and metformin + risperidone)*	**Morris water maze test:** ↓ latency to find the hidden platform, latency to reach target quadrant and ↑ time spent in target quadrant *(metformin and metformin + risperidone)* **Tail immersion nociceptive test:** ↓ tail withdrawal latency *(metformin and metformin + risperidone)*
**Mirza & Sharma**, [76]	Prenatal valproic acid (VPA)-induced, Wistar rats	Pioglitazone (*p.o*., 10 or 20 mg/kg, 28 d)	**3‑chamber test:** ↑ sociability index and social novelty preference index - ↑ sociability and social novelty preference *(10 and 20 mg/kg)*	**Y-maze test:** ↑ number of repetitive behaviors *(10 and 20 mg/kg)*	**Elevated plus maze test:** ↑ time in open arm and number of open arms entries *(10 and 20 mg/kg)* **Hole board exploration test:** ↑ number of hole poke and rearing; ↓ latency of first poke *(10 and 20 mg/kg)* **Locomotor activity on actophotometer:** ↓ number of counts *(10 and 20 mg/kg)*	Not assessed
**Sandhu et al**., [77]	Prenatal valproic acid (VPA)-induced, Wistar rats	Pioglitazone (*s.c*., 2.5, 5, 10 mg/kg, 30 d)	**3‑chamber test:** ↑ time in social or social novelty chamber - ↑ sociability and social novelty preference, ↑ sniffing time with stranger *(10 mg/kg)*	**Marble burying test:** ↓ number of buried marbles *(5 and 10 mg/kg)*	**Open field test:** ↑ time in center *(10 mg/kg)* **Elevated plus maze test:** ↑ time in open arm, ↓ time in closed arm *(10 mg/kg)* **Locomotor activity on actophotometer:** ↓ vertical and horizontal counts *(5 and 10 mg/kg)*	**T-maze test:** ↑ % alternation *(10 mg/kg)*
**Wang et al**., [74]	BTBR mice	Metformin (*i.p*., 200 mg/kg, 8 d)	**3‑chamber test:** ↑ time in social chamber and social sniffing time - ↑ sociability	**Self‑grooming test:** ↓ self-grooming time **Marble burying test:** ↓ number of buried marbles	**Open field test:** ↔ time in center and distance travelled **Elevated plus maze test:** ↔ time in open arms **Light-dark box test:** ↔ time in dark box and number of transitions	Not assessed
***Purines***						
**Hirsch et al**., [88]	Prenatal valproic acid (VPA)-induced, Wistar rats	Suramin (purinergic antagonist, *i.p*., 20 mg/kg)	**3‑chamber test:** ↑ time in social chamber and sniffing - ↑ sociability, ↑ time exploring the novel rat - ↑ social novelty preference **Reciprocal social interaction test:** ↔ total time and number of interactions, nose-to-nose sniffing, anogenital inspection and flank exploration; ↑ number of following	**Open field test:** ↔ self‑grooming number and time	**Elevated plus maze test:** ↑ time in open arm, ↔ number of open arms entries and risk assessments **Open field test:** ↑ time in central square, ↔ number of rearings, distance travelled, average speed	**Whisker nuisance test:** ↔ total score **Tail flick test:** ↔ latency to tail withdrawal
**Naviaux et al**., [86]	Maternal immune activation (MIA) - gestational poly(IC) exposure, C57BL/6 J mice	Suramin (purinergic antagonist, *i.p*., 10 or 20 mg/kg; weekly 3x)	**3‑chamber test:** ↑ time in social chamber and social sniffing - ↑ sociability	Not assessed	**Rotarod test:** ↓ latency to fall	Not assessed
**Naviaux et al**., [87]	Maternal immune activation (MIA) - gestational poly(IC) exposure, C57BL/6 J mice	Suramin (purinergic antagonist, *i.p*., 20 mg/kg)	**3‑chamber test:** ↑ time in social chamber - ↑ sociability	Not assessed	**T-maze test:** ↑ novelty preference (% of spontaneous alternation)	**Rotarod test:** ↔ latency to fall
**Vitamins**						
**Du et al**., [89]	Prenatal valproic acid (VPA)-induced, Wistar rats	Vitamin D3 (*i.m*., 80,000 IU/kg)	**Olfactory habituation/dishabituation test:** ↑ sniffing time of saline, swipes and urine **Reciprocal social interaction test:** ↓ latency of pinning, ↔ frequency of pinning	**Self‑grooming test:** ↓ self‑grooming time	Not assessed	Not assessed
**Luo et al**., [90]	Prenatal valproic acid (VPA)-induced, Sprague Dawley rats	Retinoic acid (*p.o*., 6 mg/kg, 21 d)	**3‑chamber test:** ↑ time in social or social novelty chamber - ↑ sociability and social novelty preference	**Self‑grooming test:** ↓ self-grooming time	**Open field test:** ↑ time in center	Not assessed
**Zhu et al**., [91]	Prenatal valproic acid (VPA)-induced, Sprague Dawley rats	Retinoic acid (*p.o*., 1 mg/kg, 10 d)	**3‑chamber test:** ↑ time in social novelty chamber and total social novelty score - ↑ social novelty preference	Not assessed	**Open field test:** ↔ time in center and distance travelled	Not assessed
***Others (calcium, adrenergic receptor, histone modifications)***						
**Alhamami et al**., [101]	Prenatal valproic acid (VPA)-induced, Sprague Dawley rats	6-hydroxydopamine (6‑OHDA, *i.c.v*. into CV4; 75 µg/µl, 2 d) ± LPS (*i.p*., 500 µg/kg)	Not assessed	**Self‑grooming test:** ↑ self-grooming time in 6-OHDA + LPS group *vs* VPA group	Not assessed	Not assessed
**Carreno-Muñoz et al**., [98]	*Fmr1*-KO mice	BMS-204352 (BKCa agonist, *i.p*., 2 mg/kg)	Not assessed	**Self‑grooming test:** ↓ back self-grooming time	**Open field test:** ↓ distance traveled, ↓ time in center	**Nest building test:** ↑ nest score
**Feng et al**., [100]	Prenatal valproic acid (VPA)-induced, Sprague Dawley rats	Calcium hexacyanoferrate (III) nanocatalysts (CaH NCs, *i.c.v*., 0.2 mg/kg)	**3‑chamber test:** ↑ time in social or social novelty chamber - ↑ sociability and social novelty	**Y‑maze test:** ↑ spontaneous alternation rate	**Elevated plus maze test:** ↑ number of entries and time in open arm **Open field test:** ↑ time in center and distance travelled	**Morris water maze test:** ↓ latency to find the hidden platform
**Rapanelli et al**., [99]	*Shank3*^*+/ΔC*^ mice; *Shank3*^*E13*^ mice; *Cul3*^*f/−*^ mice	GSK-LSD1 (LSD1 inhibitor, *i.p*., 5 mg/kg, 3 d); ORY-1001 (LSD1 inhibitor, *i.p*., 0.015 mg/kg, 3 d); AAV2-CMV-EGR1-Flag (Egr1 AAV into PFC, *i.c.v*., 0.3 μl per hemisphere)	*Shank3*^*+/ΔC*^ mice **3‑chamber test:** ↑ sociability index *(GSK-LSD1 and ORY-1001 or* Egr1 AAV*)*	*Shank3*^*+/ΔC*^ mice **Self‑grooming test:** ↓ self-grooming time *(GSK-LSD1)*; ↔ self-grooming time *(ORY-1001 or* Egr1 AAV*)*	*Shank3*^*+/ΔC*^ mice **Open field test:** ↔ distance travelled and time in center *(GSK-LSD1 or* Egr1 AAV*)* **Rotarod test:** ↔ latency to fall *(GSK-LSD1)*	*Shank3*^*+/ΔC*^ mice Not assessed
			*Shank3*^*E13*^ mice **3‑chamber test:** ↑ sociability index *(GSK-LSD1)*	*Shank3*^*E13*^ mice **Self‑grooming test:** ↓ self-grooming time *(GSK-LSD1)*	*Shank3*^*E13*^ mice Not assessed	*Shank3*^*E13*^ mice Not assessed
			*Cul3*^*f/−*^ mice **3‑chamber test:** ↑ sociability index *(GSK-LSD1)*	*Cul3*^*f/−*^ mice Not assessed	*Cul3*^*f/−*^ mice **Open field test:** ↔ time in center *(GSK-LSD1)*	*Cul3*^*f/−*^ mice Not assessed

↑ significant increase/improvement in the measured behavior, ↓ significant decrease/reduction in the measured behavior, ↔ no significant change, *CFU* colony-forming units, *d* days, *i.c.v*. intracerebroventricular administration, *i.m*. intramuscular administration, *i.p*. intraperitoneal administration, *p.o*. oral administration, *PAM* positive allosteric modulator, *PFC* prefrontal cortex, *SNpc* substantia nigra pars compacta.

Attempts to rescue repetitive behaviors with a single exposure to oxytocin have yielded divergent outcomes, including a positive effect in rats in the VPA model [41], no effect in *Oprm1*-KO mice [42], or even an exacerbation of these behaviors in C58/J mice, manifested by prolonged self-grooming [38]. Regardless of the dosage regimen and ASD model applied, oxytocin did not affect the locomotor activity of the tested rodents in the vast majority of studies that evaluated this aspect [33, 34, 38, 40]. The exception was observed in BALB/cByJ mice, where oxytocin administration reduced the distance traveled in the open field test [39].

Subchronic or chronic oxytocin treatment improved social behaviors in both environmental and genetic models of ASD. Enhanced sociability was reported in rats prenatally exposed to VPA [31], in inbred mouse strains with inherently low sociability, such as BALB/cByJ and C58/J mice [38, 39], and in selected genetic models, including 15q dup mice [34] and *Oprm1-*KO mice [42]. Remarkably, these beneficial effects persisted for up to two weeks following the final administration of oxytocin [38]. However, the same intervention proved ineffective in enhancing sociability in *16p11.2*^*+/−*^, *Fmr1*-KO, and *Shank3*-KO mice [40]. Repeated oxytocin administration effectively reduced repetitive behaviors such as self-grooming in VPA-exposed rats [31], but not in *16p11.2*^*+/*−^ and *Shank3*-KO mice [40]. *Fmr1*-KO mice even increased the number of self-grooming episodes after oxytocin treatment [40]. An additional effect of oxytocin administered for 7 to 28 days was a reduction in anxiety-like behaviors in rats prenatally exposed to VPA or in *16p11.2*^*+/*−^ mice [31, 40].

In addition to the native form of oxytocin, the therapeutic potential of its analogues and metabolites has been investigated. Two synthetic OXTR agonists, TC-OT-39 and carbetocin, and the metabolite [Cyt6]OT(5–9), did not increase the duration of social interaction in the BALB/cByJ mice. However, TC-OT-39 reduced repetitive behaviors in the marble burying test. In contrast, another oxytocin metabolite, [pGlu4,Cyt6]OT(4–9), increased sociability dose-dependently following subchronic administration, with effects persisting up to 12 days post-treatment. Unlike oxytocin, acute administration of [pGlu4,Cyt6]OT(4–9) did not normalize marble-burying behavior [39].

Oxytocin receptor antagonists, typically used as a pharmacological tool to confirm the mechanisms of compounds with putative prosocial activity, have provided additional insights. In *Nlgn3*-KO mice, administration of the OXTR antagonist L-368,899 reduced social interactions, confirming that inhibition of oxytocinergic signaling impairs sociability [37]. However, a study utilizing female offspring rats exposed to VPA during pregnancy yielded surprising therapeutic results with the administration of the chronic OXTR antagonist, atosiban. This treatment significantly alleviated deficits in social interaction, anxiety, and repetitive behaviors. The effectiveness of this therapeutic approach was most likely because the female rats studied were characterized by excessive activation of the oxytocin system with increased levels of both oxytocin and its receptor in the hippocampus and prefrontal cortex (PFC) [43]. Conversely, in *Oprm1* mutant mice, a single administration of the OXTR antagonist LI183 had no measurable effect on social contact duration during the reciprocal social interaction test [42].

Due to chemical similarities and cooperative roles in regulating social behavior, aggression, and learning, another neurohormone, vasopressin, has also been investigated. Its chronic intranasal administration increased sociability and social novelty preference in VPA-exposed rats [44]. Comparable effects were observed following intracerebral administration of vasopressin in *Oxtr* null mice, where the treatment also reduced aggressive behavior. In contrast, the compound SR49059, a V1a receptor antagonist, did not significantly affect social or aggressive behaviors in *Oxtr*-KO mice [33].

Some studies have also attempted to link the efficacy of pharmacological interventions targeting oxytocin signaling in normalizing ASD-like behaviors with alterations in the central nervous system. Among the main molecular mechanisms in this context, the direct effect of oxytocin administration on the activity of the signaling pathway modulated by this “social hormone” should be noted. Dai and colleagues (2018) reported that repeated intranasal administration of oxytocin led to a significant increase in oxytocin-immunoreactive cells in the paraventricular nucleus in the VPA-exposed rat model [31]. Furthermore, even a single treatment with oxytocin in rats in this preclinical model exerted a neuroprotective effect by reducing the expression of necroptosis markers such as MLKL in males and females within the hippocampus and amygdala, and RIP3 in females in the amygdala, suggesting mechanisms involving the influence of cell survival and modulation of anti-inflammatory processes [41]. In turn, *Oprm1*-KO mice treated with oxytocin exhibited altered expression levels of genes encoding oxytocin, vasopressin, and dopamine receptors within the CPu, NAc, VP/Tu, MeA, and CeA regions (for details, see Table 2) [42]. In contrast, neuroimaging studies using MRI did not show a significant effect of chronic oxytocin administration on the normalization of volume changes in the analyzed brain areas in *16p11.2*^*+/*−^, *Fmr1*-KO, and *Shank3*-KO mice [40]. In the case of intranasal arginine vasopressin treatment in VPA-exposed rats, the observed beneficial behavioral effects may be associated with its influence on hippocampal gene expression related to oligodendrocyte development and myelination (*Mbp*, *Plp1*, *Cnp*, *Gfap*, *Taok1*) [44].

**Table 2:** Summary of molecular changes in cortex, hippocampus, and other brain regions after pharmacological and microbiota-based interventions in rodent models of ASD.

Reference	Animal model	Intervention	Cortical structures	Hippocampus	Other brain regions
***Oxytocin signaling***					
**Bao et al**., [44]	Prenatal valproic acid (VPA)-induced, Wistar rats	Arginine vasopressin (*intranasal*, 400 µg/kg, 22 d)	Not assessed	**RNA-seq:** ↓ abundance of *Cnp*, *Mog*, *Mag*, *Myo1d*, *Cldn11*, *Gjc2*, *Ninj2*, *Ago3*, *Taok1*; ↑ abundance of *Mgam*, *Itga7* **BRETIGEA cell-type analysis:** ↓ oligodendrocyte proportion ↓ oligodendrocyte precursor cells **RT-qPCR:** ↓ mRNA levels of *Mbp*, *Plp1*, *Cnp*, *Gfap*, *Taok1*	Not assessed
**Dai et al**., [31]	Prenatal valproic acid (VPA)-induced, Wistar rats	Oxytocin (*intranasal*, 20 µg; *s.c*., 3 µg, 7 d)	Not assessed	Not assessed	**Immunohistochemistry:** ↑ oxytocin-immunoreactive cell number in paraventricular nucleus ↔ oxytocin-immunoreactive cell number in supraoptic nucleus
**Hörnberg et al**., [37]	*Nlgn3*-KO mice; *Fmr1*-KO mice	ETC-168 (*p.o*., 5 mg/kg, acute or 8-11 d); L-368,899 (oxytocin receptor antagonist, *i.p*., 10 mg/kg)	Not assessed	Not assessed	*Nlgn3*-KO mice **Electrophysiology:** ↔ firing frequency at baseline in dopaminergic neurons in VTA *(ETC-168)* **FUNCAT:** ↓ AHA incorporation in VTA *(ETC-168)* Global proteome analysis using TMT: ↔ protein expression of DAT, TH and Ddc
**Lindenmaier et al**., [40]	*16p11.2*^+/−^ mice; *Fmr1*-KO mice; *Shank3*-KO mice	Oxytocin (*intranasal*, 0.15 μg/10 μL, 28 d)	***In-vivo*** **and** ***ex-vivo*** **MRI: ↔** neuroanatomy (volume)	***In-vivo*** **and** ***ex-vivo*** **MRI: ↔** neuroanatomy (volume)	***In-vivo and ex-vivo*** **MRI: ↔** neuroanatomy (volume)
**Liu et al**., [43]	Prenatal valproic acid (VPA)-induced, Sprague Dawley rats	Atosiban (oxytocin receptor antagonist, *intranasal*, 100 μg/kg, 14 d)	Not assessed	**In-vivo electrophysiology:** ↑ value of LTP (fEPSP slope) **Golgi-cox staining:** ↓ spine density of CA1 and CA3 pyramidal neurons	Not assessed
**Moy et al**., **[**39**]**	BALB/cByJ mice	Oxytocin, (*i.p*., 1 or 2 mg/kg, acute or 4 d); TC-OT-39 (oxytocin analog, *i.p*., 30 or 50 mg/kg, acute or 4 d); [pGlu4,Cyt6]OT(4–9) (*i.p*., 1 or 2 mg/kg); [Cyt6] OT(5–9) (*i.p*., 1 or 2 mg/kg); Carbetocin (*i.p*., 3, 6, 10, 15, 20 mg/kg)	Not assessed	Not assessed	Not assessed
**Nagano et al**., [34]	*15q dup* mice	Oxytocin (*s.c*., 0.2-0.26 mg/kg, 15 d) 8OH-DPAT (5-HT_1A_ receptor agonist; *s.c*., 0.5 mg/kg, 15 d)	Not assessed	Not assessed	Not assessed
**Pantouli et al**., [42]	*Oprm1-*KO mice	Oxytocin (*intranasal*, 0.3 IU ∼800 μg/kg μg/kg, 6 d)	Not assessed	Not assessed	**RT-qPCR:** ↓ mRNA level of *Adora2a, Avpr1a, Drd1a, Kcc2, Oxtr, Pdyn Tac1* in the CPu; ↓ mRNA level of *Arc*, *Avpr1b, Drd1a, Drd2, Tac1* in the NAc ↓ mRNA level of *Adora2a, Avpr1a, Drd1a, Drd2, Kcc2, Oxtr* in the VP/Tu **↔** mRNA level of *evaluated genes* in the LS ↓ mRNA level of *Avp*, *Avpr1a, Drd2, Kcc2, Oxt, Oxtr, Pdyn, Tac1* in the MeA ↑ mRNA level of*, Pdyn, Penk* in the CeA
**Sala et al**., [33]	*Oxtr*-KO mice	Oxytocin (*i.c.v*., 0.5 ng); Vasopressin (*i.c.v*., 0.5 ng); SR49059 (V1a receptor antagonist, (*i.c.v*., 0.5 ng)	Not assessed	Not assessed	Not assessed
**Shariatpanahi et al**., [41]	Prenatal valproic acid (VPA)-induced, Wistar rats	Oxytocin (*intranasal*, 1 μg/μL, 10 μL per nostril)	Not assessed	**Western blot:** ↓ expression of MLKL in male and female, **↔** expression of RIP3 in male and female	*Amygdala* **Western blot:** ↓ expression of MLKL in male and female, **↔** expression of RIP3 in male, ↓ expression of RIP3 in female
**Teng et al**., [38]	BALB/cByJ mice; C58/J mice	Oxytocin (*i.p*., 1 mg/kg, 1 d or 4 d)	Not assessed	Not assessed	Not assessed
***Neurotrasmission (GABA, glutamete, DA, 5-HT)***					
**Chao et al**., [60]	BTBR mice; *Fmr1*-KO mice	Dopamine hydrochloride (*intranasal*, 3 mg/kg)	*BTBR mice* Not assessed	*BTBR mice* Not assessed	*BTBR mice* **Western blot:** ↑ expression of TH in striatum, **↔** expression of DAT in striatum
			*Fmr1*-KO *mice* Not assessed	*Fmr1*-KO *mice* Not assessed	*Fmr1*-KO *mice* **Western blot:** ↓ expression of TH in striatum **↔** expression of DAT in striatum
**D’Addario et al**., [57]	*Fmr1*-KO mice	PD158780 (ErbB inhibitor, *i.c.v*. into SNpc, 10 µM or *i.p*., 10 mg/kg)	Not assessed	Not assessed	Substantia nigra pars compacta (SNpc) **Electrophysiology:** ↓ IDHPG amplitudes, spontaneous firing frequency and number of action potentials (reduction of mGluR1 function)
**Derieux et al**., [55]	*Oprm1-*KO mice	NaBr (*i.p*., 250 mg/kg, 15 d)	Not assessed	Not assessed	**RT-qPCR:** ↓ mRNA level of *ClCa1*, ↑ mRNA level of *Gabra2*, *Grm4*, *Oxt* in the NAc ↑ mRNA level of *ClCa1*, *Oxt*, **↔** mRNA level of *Gabra2* in the VP/Tu ↑ mRNA level of *ClCa1*, *Gabra2*, *Gabra3*, *Gabra4*, *Gabra5*, *Gabrg1* and *Gabrb2* in the CPu ↑ mRNA level of *Oxt*; **↔** mRNA level of *ClCa1*, *Gabra2* in the MeA ↑ mRNA level of *Oxt*; **↔** mRNA level of *ClCa1* in the VTA/SNc
**Dobrovolsky et al**., [50]	Valproic acid (VPA)-induced, Wistar rats (exposure pups)	Xenon (25% inhalation, 10 min)	Not assessed	Not assessed	Not assessed
**Habib et al**., [53]	Prenatal valproic acid (VPA)-induced, Wistar rats	Risperidone (*i.p*., 1 or 3 mg/kg, 28 d)	*Prefrontal cortex* **RT-qPCR:** ↑ mRNA level of *Adar2*, ↓ *GluA2* transcript Q:R *(1 and 3 mg/kg)* **ELISA:** ↓ level of cytochrome-c, LDH *(1 and 3 mg/kg)* and caspase-3 *(3 mg/kg)* **Colorimetric assay:** ↑ level of total antioxidant capacity (TAC), reduced glutathione (GSH); ↓ level of malondialdehyde (MDA) and nitric oxide (NO) **Immunohistochemistry:** ↑ number of viable neurons, optical density of toluidine blue and BCL2 positive cells; ↓ number of caspase-3 positive cells *(1 and 3 mg/kg)*	**RT-qPCR:** ↑ mRNA level of *Adar2*, ↓ G*luA2* transcript Q:R *(1 and 3 mg/kg)* **ELISA:** ↓ level of cytochrome-c, LDH and caspase-3 *(1 and 3 mg/kg)* **Colorimetric assay:** ↑ level of total antioxidant capacity (TAC), reduced glutathione (GSH); ↓ level of malondialdehyde (MDA) and nitric oxide (NO) (*1 and 3 mg/kg)* **Immunohistochemistry**: ↑ number of viable neurons, optical density of toluidine blue and BCL2 positive cells in CA1 and DG; ↓ number of caspase-3 positive cells in CA1 and DG *(1 and 3 mg/kg)*	Not assessed
**Rahdar et al**., [62]	Prenatal valproic acid (VPA)-induced, Wistar rats	LP-211 (5-HT_7_ receptor agonist, 10 nmol/l, brain slices bath)	Not assessed	*CA1 pyramidal neurons* **Electrophysiology** ***ex vivo*:** reverse electrophysiological abnormalities, e.g.: ↓ spontaneous firing frequency and input resistance; ↑ time constant, membrane capacitance and sag ratio, rheobase currents and utilization time; adaptation index and coefficient of variation; latency of onset; **↔** resting membrane potential	Not assessed
**Santrač et al**., [58]	Prenatal valproic acid (VPA)-induced, Wistar rats	MP-III-022 (α5GABAAR PAM, *i.p*., 0.33 or 1 mg/kg, 7 d); 30 nM MP-III-022 in vitro	Not assessed	**RT-qPCR:** ↑ mRNA level of *KCC2* in female (*1 mg/kg)*, ↔ mRNA level of *KCC2* in male and *Gabra5*, *NKCC1* in male and female **Intracellular calcium imaging in vitro:** ↑ amplitude of spontaneous calcium oscillations	Not assessed
**Tu et al**., [52]	*Mef2c*^*+/−*^ mice	NitroSynapsin (*i.p*., 4.6 μmol/kg, twice a day for 90 d)	Not assessed	**Immunohistochemistry:** ↑ number of NeuN positive cells and level of VGAT immunoreactivity, level of parvalbumin-positive synapses immunoreactivity, **↔** level of VGLUT1 immunoreactivity, parvalbumin positive cells, ↓ level of VGLUT2 immunoreactivity, VGLUT1/VGAT ratio and VGLUT2/VGAT ratio, number of GFAP positive cells; number of cell counts in TUNEL staining and activated caspase-3 **Electrophysiology** ***ex vivo*:** ↑ fEPSP slope	Not assessed
**Vicidomini et al**., [54]	*Shank3*^*Δ11 -/-*^ mice	CDPPB (mGlu5 receptor PAM, *i.p*., 3 mg/kg, acute/chronic)	Not assessed	Not assessed	Not assessed
**Yoshimura et al**., [56]	BTBR mice	GRN-529 (*i.p*., 3 mg/kg); 2-261 (*i.p*., 0.1, 0.3, 1, 3 mg/kg); 2-301 (*i.p*., 0.3, 1, 3 mg/kg); 2-313 (*i.p*., 1, 3 mg/kg); AVL-3288 (*i.p*., 0.3, 1, 3 mg/kg); 4-327 (*i.p*., 1, 3 mg/kg)	Not assessed	Not assessed	Not assessed
**Zohny et al**., [51]	Prenatal valproic acid (VPA)-induced, Wistar rats	Memantine (*i.p*., 20 mg/kg, 38 d); Aripiprazole (*i.p*., 3 mg/kg, 38 d); Memantine (*i.p*., 20 mg/kg, 38 d) + Aripiprazole (*i.p*., 3 mg/kg, 38 d)	Not assessed	**HPLC:** ↓ level of glutamate; ↑ level of GABA and GABA/glutamate ratio *(memantine, aripiprazole, memantine + aripiprazole)* **RT-qPCR:** ↑ mRNA level of *Glt-1 (memantine, aripiprazole, memantine + aripiprazole)* **Western blot: ↔** expression of total CREB, ↑ expression of p-CREB *(memantine + aripiprazole)*, ↑ expression of BDNF *(memantine, aripiprazole, memantine + aripiprazole)*, **Immunohistochemistry:** ↑ number of intact neurons, Nissl’s granules density, level of BCL-2 immunoreactivity in CA1 and DG *(memantine, aripiprazole, memantine + aripiprazole)*, ↓ number of NFTs, level of GFAP, Caspase-3 and BAX immunoreactivity in CA1 and DG *(memantine, aripiprazole, memantine + aripiprazole)*	Not assessed
***Anti-inflammatory***					
**Abdel-Haq et al**., [95]	*Shank3*^*Δ4-22*^ mice	7‑NI (nNOS inhibitor; *s.c*., PSARA gel formulation, 80 mg/kg)	Not assessed	Not assessed	Not assessed
**Cristiano et al**., [93]	BTBR mice; Prenatal valproic acid (VPA)-induced, B6 mice	MR‑39 (FPR2 agonist; *i.p*., 10 mg/kg, 8 d); In vitro*:* MR-39 (10 µM for 4 or 72 h)	*BTBR mice* Not assessed	*BTBR mice* **RT-qPCR:** ↑ mRNA level of *Fpr2, Il-10;* ↓ mRNA level of *Il-1β*, *Tnf-α* **Western blot:** ↑ expression of FPR2 **ELISA:** ↑ level of LX4A **In vitro*:*** ↑ neurite length	*BTBR mice* Not assessed
			*Prenatal valproic acid (VPA)-induced, B6 mice* Not assessed	*Prenatal valproic acid (VPA)-induced, B6 mice* **RT-qPCR:** ↑ mRNA level of *Fpr2, Il-10;* ↓ mRNA level of *Il-1β*, *Tnf-α* **Western blot: ↔** expression of FPR2 **ELISA:** ↑ level of LX4A	*Prenatal valproic acid (VPA)-induced, B6 mice* Not assessed
**Erdoğan et al**., [96]	Propionic acid (PPA)-induced, male Wistar rats	Pentoxifylline (*p.o*., 300 mg/kg, 15 d)	Not assessed	**Immunohistochemistry:** ↑ neuronal density in CA1 and CA3, ↓ level of GFAP immunoreactivity in CA1, **↔** level of GFAP immunoreactivity in CA3	*Whole brain* **ELISA:** ↓ level of IL-17, TNF*-α;* ↑ level of ATP, NGF **TBARS method**: ↓ level of MDA *Cerebellum* **Immunohistochemistry:** ↑ number of Purkinje cells, ↓ level of GFAP immunoreactivity
**Hidema et al**., [92]	*Oxtr*-KO mice; Prenatal valproic acid (VPA)-induced, C57BL6/J mice	Resveratrol (*i.p*., 30 mg/kg)	*Oxtr*-KO mice Not assessed	*Oxtr*-KO mice Not assessed	*Oxtr*-KO mice **RT-qPCR:** ↑ mRNA level of *Egr3* and *Sirt1* in amygdala
			*Prenatal valproic acid (VPA)-induced, C57BL6/J mice* Not assessed	*Prenatal valproic acid (VPA)-induced, C57BL6/J mice* Not assessed	*Prenatal valproic acid (VPA)-induced, C57BL6/J mice* **RT-qPCR:** ↔ mRNA level of *Egr3* and *Sirt1* in amygdala
**Özkul et al**., [94]	Propionic acid (PPA)-induced, male Wistar rats	Vardenafil (*p.o*., 3 mg/kg, 15 d)	Not assessed	Not assessed	**ELISA:** ↓ level of IL-2, IL-17, lactate, TNF*-α;* ↑ level of NGF, c-GMP in whole brain **Immunohistochemistry:** ↑ neuronal density in CA1 and CA3 of hippocampus, ↑ number of Purkinje cells in cerebellum, ↓ level of GFAP immunoreactivity in CA1 and CA3 of hippocampus and cerebellum **MRI** ***in vivo*:** ↓ level of lactate in corpus striatum
**Zhang et al**., [97]	Prenatal valproic acid (VPA)-induced, Sprague Dawley rats	Aspirin (*i.p*., 1 mg/kg, 30 d)	Not assessed	**Western blot:** ↑ expression of p-ACC, p-AMPK	Not assessed
***Microbiota***					
**Abuaish et al**., [70]	Propionic acid (PPA)-induced, male Sprague Dawley rats	Fecal microbiota transplant (*p.o*., 1 g/kg/day, 22 d); *Bifidobacterium longum* BB536 (*p.o*., 1×10⁹ CFU/day, 22 d)	Not assessed	**RT-qPCR:** ↓ mRNA level of *Bdnf*, ↑ mRNA level of *Mecp2*, *Slc17a7 (FMT)* ↔ mRNA level of *Bdnf*, *Mecp2*, *Slc17a7 (B. longum) and Gad1 (FMT, B. longum)*	Not assessed
**Abujamel et al**., [66]	Propionic acid (PPA)-induced, male Sprague Dawley rats	Fecal microbiota transplant (*p.o*., 1 g/kg/day, 30 d); *Bifidobacterium longum* BB536 (*p.o*., 1 × 10⁹ CFU/day, 30 d)	Not assessed	Not assessed	Not assessed
**Kong et al**., [67]	Prenatal valproic acid (VPA)-induced, Wistar rats	*Bifidobacterium longum* CCFM1077 (*p.o*., 10⁹ CFU/mL); Risperidone (*p.o*., 1.6 g/100 g)	**HPLC:** ↔ level of GABA, DA and Glu/GABA ratio *(B. longum, risperidone)*, ↓ level of Glu *(risperidone)*, ↑ level of NE *(B. longum, risperidone)* and ACH *B. longum)* in prefrontal cortex	Not assessed	**HPLC:** ↑ level of tryptophan (TRP), ↓ kynurenine/TRP ratio *(risperidone)* ↔ level of TRP (*B. longum*), kynurenine, kynurenic acid *(B. longum, risperidone)* ↓ level of quinolinic acid (*B. longum)* ↔ level of quinolinic acid *(risperidone)* ↑ level of GABA *(B. longum, risperidone) and NE (risperidone)*, ↓ level of Glu (*B. longum)* and Glu/GABA ratio *(B. longum, risperidone)*, ↔ level of DA and ACH *(B. longum, risperidone)* in cerebellum
**Mintál et al**., [68]	Prenatal valproic acid (VPA)-induced, Wistar rats	Probiotic mixture (*Lactobacillus spp., Bifidobacterium spp*; *p.o*., 14 d)	Not assessed	**Immunohistochemistry:** diameter of the various hippocampal regions - data not to be correctly interpretable	Not assessed
**Wang et al**., [71]	Prenatal valproic acid (VPA)-induced, C57BL6/J mice Prenatal valproic acid (VPA)-induced, C57BL6/J mice + Fecal microbiota transplantation from ASD patients	Fecal microbiota transplantation from healthy patients (*p.o*., 21 d)	**RNA-seq:** 81 upregulated genes and 141 downregulated genes (calcium signaling pathway, MAPK signaling pathway, and important neurotransmitter systems, including glutamatergic synapse, serotonergic synapse, and GABAergic synapse) were identified in the VPA_TD_FMT group versus the VPA_ASD_FMT group	**Immunohistochemistry:** ↑ level of tyrosine hydroxylase immunoreactivity	Not assessed
**Wang et al**., [69]	Prenatal valproic acid (VPA)-induced, C57BL6/J mice	*Lactobacillus reuteri C501* (*p.o*., 10^8^ CFU, 28 d); Inulin (*p.o*., 1% w/v in drinking water, 28 d); Synbiotic (*p.o*., *L. reuteri C501*, 10^8^ CFU + inulin, 1% w/v, 28 d)	**RT-qPCR:** ↓ mRNA level of *Il-6, Tnf-α*, ↔ mRNA level of *Bdnf, Gpr41, Gpr109a, Il-1β, Il-10, Mct1, Mecp2, Psd95, Pten, Snap25(L. reuteri)* ↑ mRNA level of *Gpr41, Gpr109a, Pten, Snap25*, ↓ mRNA level of *Il-6, Tnf-α*, ↔ mRNA level of *Bdnf, Il-1β, Il-10, Mct1, Mecp2, Psd95 (inulin)* ↑ mRNA level of *Bdnf, Gpr41, Gpr109a, Il-10, Mct1, Mecp2, Psd95, Pten, Snap25*, ↓ mRNA level of *TNF-α, Il-1β, Il-6 (synbiotic)* **Immunohistochemistry:** ↑ level of BDNF and NeuN immunoreactivity, ↓ level of Iba1 and GFAP immunoreactivity *(L. reuteri, inulin, synbiotic)* ↑ number of Nissl positive cells *(synbiotic)*	Not assessed	Not assessed
***Cannabinoids***					
**Loomis et al**., [85]	Prenatal valproic acid (VPA)-induced, Wistar rats	JZP541 (*i.p*., acute 100 mg/kg or 10, 30, 100 mg/kg, 5 d); Risperidone (*i.p*., 0.125 mg/kg, 5 d)	*Frontal cortex* **RNA-seq:** 6 upregulated genes and 1 downregulated genes *(10 mg/kg)*, 67 upregulated genes and 33 downregulated genes *(30 mg/kg)* 785 upregulated genes and 548 downregulated genes *(100 mg/kg)*	**RNA-seq:** 2 upregulated genes and 1 downregulated genes *(10 mg/kg)*, 12 upregulated genes and 12 downregulated genes *(30 mg/kg)* 437 upregulated genes and 414 downregulated genes *(100 mg/kg)*	Not assessed
**Pedrazzi et al**., [83]	Prenatal valproic acid (VPA)-induced, Swiss mice	Cannabidiol (*i.p*., 30 or 60 mg/kg)	Not assessed	Not assessed	Not assessed
**Poleg et al**., [82]	*InsG3680 Shank3* mice	Avidekel oil (*p.o*., 5 ml/kg, (CBD:THC ratio 20:1, 25 mg/kg CBD, 1 mg/kg THC, twice a week for 21 d))	Not assessed	Not assessed	*Cerebellum* **RNA-seq:** changes in abundance of several autism-related genes, such as *Aqp4*, *Dync1h1*, *Neo1*, *Dbh*, genes coding for ion channels such as *Scn2b*, *Scn8a*, *Kcna2*, heat shock proteins such as *Hspa1a*, *Hspa1b*, genes regulating the membrane potential, action potential and transmission of nerve impulse
**Zamberletti et al**., [84]	Prenatal valproic acid (VPA)-induced, Sprague Dawley rats	Cannabidivarin (*i.p*., 20 mg/kg, 23 d – symptomatic schedule)	Prefrontal cortex **Western blot: ↔** expression of CB1 receptor, CB2 receptor, CD11b, DAGLα, FAAH, GFAP, MAGL, NAPE-PLD, TNF-α	**Immunohistochemistry:** ↑ soma roundness, surface area, perimeter of microglia cells, ↓ soma area of microglia cells **Western blot:** ↓ expression of CB1 receptor, GFAP, MAGL, TNF-α **↔** expression of CB2 receptor, CD11b, DAGLα, FAAH, NAPE-PLD	Not assessed
***Metabolic***					
**Elnahas et al**., [75]	Prenatal valproic acid (VPA)-induced, Wistar rats	Metformin (*i.p*., 100 mg/kg, 38 d); Risperidone (*i.p*., 1 mg/kg, 38 d); Metformin (*i.p*., 100 mg/kg, 38 d) + Risperidone (*i.p*., 1 mg/kg, 38 d)	*Prefrontal cortex* **ELISA:** ↓ level of IL-1β, NF-κB, TNF-α *(metformin, risperidone, metformin+risperidone)* **Colorimetric assay:** ↓ level of MDA, NO_x_, ↑ level of GSH *(metformin, risperidone, metformin+risperidone)*, ↑ level of CAT *(risperidone, metformin+risperidone)* **RT-qPCR:** ↑ mRNA level of *Ppar-α (metformin, risperidone, metformin+risperidone)* **Immunohistochemistry:** ↑ number of viable neurons, Nissl’s granules density, ↓ number of NFTs, level of caspase-3 and GFAP immunoreactivity *(metformin, risperidone, metformin+risperidone)*	**ELISA:** ↓ level of IL-1β, NF-κB, TNF-α *(metformin, risperidone, metformin+risperidone)* **Colorimetric assay:** ↓ level of MDA, NO_x_, ↑ level of CAT, GSH *(metformin, risperidone, metformin+risperidone)* **RT-qPCR:** ↑ mRNA level of *Ppar-α (metformin, risperidone, metformin+risperidone)* **Immunohistochemistry:** ↑ number of viable neurons, Nissl’s granules density, ↓ number of NFTs, level of caspase-3 and GFAP immunoreactivity *(metformin, risperidone, metformin+risperidone)*	Not assessed
**Mirza & Sharma**, [76]	Prenatal valproic acid (VPA)-induced, Wistar rats	Pioglitazone (*p.o*., 10 or 20 mg/kg, 28 d)	*Prefrontal cortex* **ELISA:** ↑ level of IL-10, ↓ level of IL-6, TNF-α **Colorimetric assay:** ↑ level of GSH, ↓ level of TBARS	Not assessed	*Cerebellum* **ELISA:** ↑ level of IL-10, ↓ level of IL-6, TNF-α **Colorimetric assay:** ↑ level of GSH, ↓ level of TBARS *Brainstem* **ELISA:** ↑ level of IL-10, ↓ level of IL-6, TNF-α **Colorimetric assay:** ↑ level of GSH, ↓ level of TBARS
**Sandhu et al**., [77]	Prenatal valproic acid (VPA)-induced, Wistar rats	Pioglitazone (*s.c*., 2.5, 5, 10 mg/kg, 30 d)	*Prefrontal cortex* **Colorimetric assay:** ↑ level of GSH, catalase activity, ↓ level of nitrite *(10 mg/kg)*, ↔ level of MDA, SOD activity *(2.5, 5, 10 mg/kg)*	**Colorimetric assay:** ↔ level of GSH, MDA, catalase activity *(2.5, 5 mg/kg)*, ↑ SOD activity, ↓ level of nitrite *(5, 10 mg/kg)* **Immunohistochemistry:** ↑ number of viable neurons in CA1 and CA3 *(10 mg/kg)* **RT-qPCR:** ↑ mRNA level of *Bcl-2*, ↓ mRNA level *Il-6*, *Tnf-α (5, 10 mg/kg)*, ↔ mRNA level *caspase-3*, *Il-10 (2.5, 5, 10 mg/kg)* **Western blot:** ↑ expression of Bcl-2 *(5, 10 mg/kg)*	*Cerebellum* **Colorimetric assay:** ↑ level of GSH, catalase and SOD activity, ↓ level of MDA *(10 mg/kg)*, nitrite *(5, 10 mg/kg)* **Immunohistochemistry:** ↑ number of Purkinje cells, number of viable neurons *(10 mg/kg)* *Brainstem* **Colorimetric assay:** ↑ catalase activity, ↓ level of MDA *(10 mg/kg)*, ↔ level of GSH, nitrite, SOD activity *(2.5, 5, 10 mg/kg)*
**Wang et al**., [74]	BTBR mice	Metformin (*i.p*., 200 mg/kg, 8 d)	**RT-qPCR:** ↓ mRNA level of *mTor*, *S6K*, ↔ mRNA level of *NF-κB* **Western blot:** ↓ expression of mTOR	Not assessed	Not assessed
***Purines***					
**Hirsch et al**., [88]	Prenatal valproic acid (VPA)-induced, Wistar rats	Suramin (purinergic antagonist, *i.p*., 20 mg/kg)	*Prefrontal cortex* **RT-qPCR:** ↔ mRNA level of purinergic receptors: *P2X3*, *P2X4*, *P2X7*, *P2Y1*, *P2Y6*, *P2Y12* and *Il-1β, Il-6, Ifn-γ, Tnf-α*	**RT-qPCR:** ↔ mRNA level of purinergic receptors: *P2X4*, *P2X7*, *P2Y1*, *P2Y2*, *P2Y6* and *Il-1β, Il-6, Ifn-γ, Tnf-α*	Not assessed
**Naviaux et al**., [86]	Maternal immune activation (MIA) - gestational poly(IC) exposure, C57BL/6 J mice	Suramin (purinergic antagonist, *i.p*., 10 or 20 mg/kg; weekly 3x)	Not assessed	Not assessed	**Western blot:** ↔ expression of core subunits of complexes I, II, III, IV, and V of mitochondrial respiratory chain ↓ activity of Mitochondrial Respiratory Chain Complex I and IV **Immunohistochemistry:** ↑ number of Purkinje cells in cerebellum
**Naviaux et al**., [87]	Maternal immune activation (MIA) - gestational poly(IC) exposure, C57BL/6 J mice	Suramin (purinergic antagonist, *i.p*., 20 mg/kg)	Not assessed	Not assessed	Not assessed
**Vitamins**					
**Du et al**., [89]	Prenatal valproic acid (VPA)-induced, Wistar rats	Vitamin D3 (*i.m*., 80,000 IU/kg)	Not assessed	Not assessed	Not assessed
**Luo et al**., [90]	Prenatal valproic acid (VPA)-induced, Sprague Dawley rats	Retinoic acid (*p.o*., 6 mg/kg, 21 d)	*Prefrontal cortex* **Western blot:** ↑ expression of ARG-1, RAR*α*, TREM2, ↓ expression of INOS **Immunofluorescence:** ↓ number of IBA-1 positive cells, ↑ number of intersections at certain distances (10–45 μm) away from the soma **RT-qPCR:** ↑ mRNA level of *Rarα, Trem2*	Not assessed	Not assessed
**Zhu et al**., [91]	Prenatal valproic acid (VPA)-induced, Sprague Dawley rats	Retinoic acid (*p.o*., 1 mg/kg, 10 d)	Not assessed	Not assessed	**BOLD-fMRI:** hypo-connectivity mainly between CP and Lec, CP and Bnst, CP and Cornu3, Cornu3 and End, Cornu3 and Gpe, Cornu3 and Vta, Vta and Bnst, Lec and End, Lec and Vta, Bnst and Gpe, and hyper-connectivity between Fn and Llc, Fn and Lld, Fn and Mld, Fn and Ent, Fn and Hypotha, Fn and Stn, Fn and Pg, Fn and St, Fn and Acp, Fn and Pg *Hypothalamus* **BOLD-fMRI:** hypo-connectivity between Hypotha and Pre, Hypotha and Den, Hypotha and Cornu 2, Hypotha and Cornu 1, Hypotha and Ll, Hypotha and Sva, Hypotha and Endo, Hypotha and Amgy, and hyper-connectivity between Hypotha and Fn **RT-qPCR:** ↓ mRNA level of *Grin2b, Nrxn1, Cacna1e, Gabrb2*, ↔ mRNA level of *Cdc42bpb, Ctnnb1, Dip2c, Gnai1, Hivep3, Kcnma1, Mecp2, Myo5a, Nr3c2, Reln, Setd5, Ski, Tanc2, Taok1, Tlk2, Ubn2* **Western blot:** ↓ expression of Grin2b, ↔ expression of Cacna1e Gabrb2, Nrxn1 **RNA-seq:** downregulation of *Gabrb2, Taok1, Mecp2, Cacna1e, Myo5a, Dip2c, Nr3c2, Ubr1, LOC680039, Nrxn1, Hivep3, Grin2b, Tanc2, Kcnma1, Setd5, Gnai1, Ubn2, Ski, Ctnnb1, Tlk2, Cdc42bpb*, *Reln, Tbl1xr1, Cask*
***Others (calcium, adrenergic receptor, histone modifications)***					
**Alhamami et al**., [101]	Prenatal valproic acid (VPA)-induced, Sprague Dawley rats	6-hydroxydopamine (6‑OHDA, *i.c.v*. into CV4; 75 µg/µl, 2 d) ± LPS (*i.p*., 500 µg/kg)	*Prefrontal cortex* **RT-qPCR:** ↑ mRNA level of *Il-6*, *Tnf-α (6-OHDA), NF-κB, (6-OHDA and 6-OHDA* + *LPS)* **Western blot:** ↓ expression of GPX-1 (*6-OHDA* + *LPS)*, ↔ expression of COX-2, AMPK, p-AMPK	**RT-qPCR:** ↔ mRNA level of *Il-6*, *NF-κB, Tnf-α* **Western blot:** ↔ expression of COX-2, GPX-1, AMPK, p-AMPK	Not assessed
**Carreno-Muñoz et al**., [98]	*Fmr1*-KO mice	BMS-204352 (BKCa agonist, *i.p*., 2 mg/kg)	Not assessed	Not assessed	Not assessed
**Feng et al**., [100]	Prenatal valproic acid (VPA)-induced, Sprague Dawley rats	Calcium hexacyanoferrate (III) nanocatalysts (CaH NCs, *i.c.v*., 0.2 mg/kg)	Not assessed	**RNA-seq:** 280 differentially expressed genes **Immunofluorescence:** ↓ level of ROS, Iba-1 and GFAP immunoreactivity **Western blot**: ↔ expression of iNOS, CD86, Arg1 (n = 3/per group)	Not assessed
**Rapanelli et al**., [99]	*Shank3*^*+/ΔC*^ mice; *Shank3*^*E13*^ mice; *Cul3*^*f/−*^ mice	GSK-LSD1 (LSD1 inhibitor, *i.p*., 5 mg/kg, 3 d)	*Shank3*^*+/ΔC*^ mice *Prefrontal cortex* **Immunofluorescence:** ↑ level of H3K4me2 immunoreactivity **Electrophysiology:** ↑ mEPSC frequency, ↔ mEPSC amplitude **RNA-seq:** 230 differentially expressed genes **RT-qPCR:** ↑ mRNA level of *Egr1, Egr4* ↔ mRNA level of *Egr3, Shank3*	*Shank3*^*+/ΔC*^ mice Not assessed	*Shank3*^*+/ΔC*^ mice Not assessed
			*Shank3*^*E13*^ mice Not assessed	*Shank3*^*E13*^ mice Not assessed	*Shank3*^*E13*^ mice Not assessed
			*Cul3*^*f/−*^ mice Not assessed	*Cul3*^*f/−*^ mice Not assessed	*Cul3*^*f/−*^ mice Not assessed

↑, significant increase; ↓, significant decrease; ↔, no significant change; *ACH*, acetylcholine; *AMPK*, AMP-activated protein kinase; *ARG-1*, arginase-1; *ATP*, adenosine triphosphate; *Avpr1a/b*, vasopressin receptor 1a/1b; *Aqp4*, aquaporine-4; *BCL-2*, B-cell lymphoma 2; *BDNF*, brain-derived neurotrophic factor; *BKCa*, large-conductance calcium-activated potassium channel; *BOLD-fMRI*, blood oxygen level–dependent functional magnetic resonance imaging; *CA1/CA3*, cornu ammonis areas 1 and 3 of the hippocampus; *CAMKII*, calcium/calmodulin-dependent protein kinase II; *CAT*, catalase; *CB1/CB2*, cannabinoid receptor type 1/type 2; *CeA*, central amygdala; *CFU*, colony-forming units; *COX-2*, cyclooxygenase-2; *CPu*, caudate putamen; *CREB*, cAMP response element-binding protein; *DA*, dopamine; *DAGLα*, diacylglycerol lipase alpha; *Dbh*, dopamine beta-hydroxylase; *DG*, dentate gyrus; *d*, days; *Dync1h1*, dynein cytoplasmic 1 heavy chain 1; *ELISA*, enzyme-linked immunosorbent assay; *fEPSP*, field excitatory postsynaptic potential; *FAAH*, fatty acid amide hydrolase; *FMRP*, fragile X mental retardation protein; *FMT*, fecal microbiota transplantation; *FPR2*, formyl peptide receptor 2; *GABA*, gamma-aminobutyric acid; *Gabra*, GABA receptor subunit alpha; *GFAP*, glial fibrillary acidic protein; *Glt-1*, glutamate transporter 1; *Glu*, glutamate; *GPX-1*, glutathione peroxidase 1; *Gpr41/109a*, G protein-coupled receptor 41/109a; *GSH*, reduced glutathione; *H3K4me2*, dimethylated lysine 4 on histone H3; *HPLC*, high-performance liquid chromatography; *Hspa1a*, heat shock protein family a member 1a; *Hspa1b*, heat shock protein family a member 1b; *IBA-1*, ionized calcium-binding adaptor molecule 1; *IDHPG*, DHPG-induced current; *IFN-γ*, interferon gamma; *IL*, interleukin; *INOS*, inducible nitric oxide synthase; *i.c.v*., intracerebroventricular administration; *i.m*., intramuscular administration; *i.p*., intraperitoneal administration; *Kcna2*, potassium voltage-gated channel subfamily a member 2; *KO*, knockout; *LPS*, lipopolysaccharide; *LS*, lateral septum; *LTP*, long-term potentiation; *MAGL*, monoacylglycerol lipase; *MAPK*, mitogen-activated protein kinase; *MDA*, malondialdehyde; *Mecp2*, methyl CpG binding protein 2; *MeA*, medial amygdala; *mEPSC*, miniature excitatory postsynaptic current; *MRI*, magnetic resonance imaging; *mRNA*, messenger RNA; *mTOR*, mechanistic target of rapamycin; *NaBr*, sodium bromide; *NAc*, nucleus accumbens; *nAchRa7*, Nicotinic Acetylcholine Receptor subunit a7; *NAPE-PLD*, N-acyl phosphatidylethanolamine phospholipase D; *NE*, norepinephrine; *Neo1*, neogenin1; *NeuN*, neuronal nuclei protein; *NF-κB*, nuclear factor kappa-light-chain-enhancer of activated B cells; *NGF*, nerve growth factor; *nNOS*, neuronal nitric oxide synthase; *NO*, nitric oxide; *Oxt*, oxytocin; *Oxtr*, oxytocin receptor; *p-CREB*, phosphorylated CREB; *PAM*, positive allosteric modulator; *PFC*, prefrontal cortex; *Ppar-α*, peroxisome proliferator-activated receptor alpha; *PPA*, propionic acid; *p.o*., oral administration; *RARα*, retinoic acid receptor alpha; *RIP3*, receptor-interacting protein kinase 3; *RNA-seq*, RNA sequencing; *ROS*, reactive oxygen species; *RT-qPCR*, reverse transcription quantitative polymerase chain reaction; *S6K*, ribosomal protein S6 kinase; *s.c*., subcutaneous administration; *Scn2b*, sodium voltage-gated channel beta subunit 2; *Scn8a*, sodium voltage-gated channel alpha subunit 8 *Sirt1*, sirtuin 1; *SNpc*, substantia nigra pars compacta; *SOD*, superoxide dismutase; *TAC*, total antioxidant capacity; *TH*, tyrosine hydroxylase; *THC*, tetrahydrocannabinol; *TNF-α*, tumor necrosis factor alpha; *TREM2*, triggering receptor expressed on myeloid cells 2; *TRP*, tryptophan; *VGAT*, vesicular GABA transporter; *VGLUT*, vesicular glutamate transporter; *VP/Tu*, ventral pallidum/olfactory tubercle; *VPA*, valproic acid; *VTA*, ventral tegmental area.

### Neurotransmission and E/I balance as therapeutic targets in ASD models

One of the leading hypotheses assumes that among the leading pathophysiological mechanisms underlying the behavioral characteristics of ASD may partly stem from an imbalance between excitatory (predominantly glutamatergic) and inhibitory (predominantly GABAergic) neurotransmission [45]. Since impairments in synaptic plasticity and network activity balance are considered key factors influencing the pathophysiology of ASD, interventions aimed at restoring typical network dynamics may impact many fundamental areas, including social behaviors as well as restrictive and repetitive behaviors, despite the high phenotypic variability observed in patients diagnosed with ASD, even within twin pairs [46–48]. Based on this assumption, several studies evaluated the efficacy of pharmacological compounds modulating key neurotransmitter systems, including glutamatergic, GABAergic, dopaminergic, serotonergic, and cholinergic signaling, to alleviate ASD-like phenotypes.

The glutamatergic system is the leading excitatory network in the brain, and the analyzed interventions focused primarily on modulating its major classes of receptors, including ionotropic *N*-methyl-D-aspartate receptors (NMDA), α-amino-3-hydroxy-5-methyl-4-isoxazolopropionic acid (AMPA), and metabotropic glutamate (mGlu) receptors [49]. Thus, short-term inhalation of subanesthetic xenon, an NMDA receptor antagonist, improved social behavior by reducing aggressive episodes and increasing social contacts in a rat model of ASD induced by VPA in the early stages of development, without affecting repetitive, anxiety-like, or depression-like behaviors [50]. Similarly, chronic treatment with memantine, an uncompetitive/fast off-rate NMDA receptor antagonist, administered alone or in combination with the atypical antipsychotic drug aripiprazole, increased sociability and social novelty preference index in the three-chamber test, while alleviating repetitive behaviors and cognitive deficits in rats prenatally exposed to VPA [51]. Furthermore, three months of treatment with NitroSynapsin, a compound exhibiting dual memantine-like action and redox-based inhibition of extrasynaptic NMDA receptors, was effective in increasing sociability in *Mef2c*^*+/−*^ mice [52]. In addition to the NMDA receptor, AMPA receptor modulation should be noted as a promising therapeutic approach in treating ASD. A recent study has revealed a new mechanism of action for the second-generation antipsychotic risperidone, whose chronic administration improves behavioral deficits (increasing sociality and social novelty preference, while reducing repetitive behaviors) caused by prenatal exposure to VPA. These beneficial effects were associated with increased expression of the ADAR2 enzyme, which promotes GluA2 subunit editing, thereby reducing excitotoxicity, oxidative stress, and neurodegeneration [53].

Positive allosteric modulators (PAMs) of mGlu receptors are effective in genetic models of ASD. In *Shank3*^*Δ11−/−*^ mice, using the mGlu5 receptor PAM - CDPPB effectively alleviated behavioral deficits, including impaired sociability and repetitive behaviors, without affecting locomotor activity [54]. In another study, treatment with an mGlu4 receptor PAM (VU0155041) exhibited a synergistic effect in combination with sodium bromide in increasing social behaviors and reducing motor stereotypies in *Oprm1*-KO mice [55]. Interestingly, administration of GRN-529, a negative allosteric modulator of the mGlu5 receptor in BTBR mice, effectively rescued an ASD-like phenotype [56]. In turn, recent work in *Fmr1*-KO mice, a model of fragile X syndrome, has indicated that the hyperactivity of dopamine neurons in the compact part of the substantia nigra is a key factor driving repetitive behaviors. This hyperactivity has been linked to the interactions between upregulated expression of ErbB tyrosine kinases and hyperfunction of the mGlu1 receptor. Consequently, inhibition of the ErbB pathway using its inhibitor PD158780 normalized mGlu1 receptor signaling, restored dopamine neuron hyperactivity, and alleviated repetitive behaviors, including reduction in self-grooming duration and marble-burying events [57].

Targeting the GABAergic system aims to enhance synaptic inhibition and correct the E/I imbalance on the inhibitory side. In a model of idiopathic ASD, BTBR mice, selective positive allosteric modulation of GABA-A receptors containing β2/3 subunits with compound 2–261 at a minimum effective dose of 0.3 mg/kg improved social behavior, although it did not affect repetitive self-grooming. Structurally related analogs demonstrated similar effects, with compound 2–301 being effective at 1–3 mg/kg doses and compound 2–313 only at the highest dose of 3 mg/kg [56]. Focusing on another receptor subunit, treatment of VPA-exposed rats with MP-III-022, a selective PAM for α5GABA-A receptors, improved social, repetitive, and restrictive behaviors in a sex-dependent manner, with more promising effects observed in males [58]. In contrast, a broader approach using chronic administration of sodium bromide, which enhances neuronal hyperpolarization by substituting chloride ions in the GABA-A receptor, was effective across three different genetic mouse models of ASD (*Oprm1*-KO, *Fmr1*-KO, and *Shank3*^*Δex13-16-/-*^ mice). This intervention effectively alleviated core symptoms, including social deficits, stereotypies, and anxiety, showing a consistently strong effect across different genetic etiologies [55].

The nicotinic cholinergic system also regulates the E/I balance and has been investigated as a potential therapeutic target. In the BTBR mice, positive allosteric modulation of the α7 nicotinic acetylcholine (nACh) receptor with AVL-3288 at higher doses (3–10 mg/kg), or the compound 4–327, proved to be effective in alleviating both deficits in social interactions and repetitive self-grooming behaviors [56].

Alterations in monoaminergic signaling have long been linked to ASD, making these systems promising therapeutic targets [59]. Studies have revealed distinct dysregulation in the dopamine system across various preclinical ASD models. The BTBR mice show an overall reduction in tyrosine hydroxylase (TH), a key enzyme in dopamine synthesis, while the *Fmr1*-KO mice show abnormal TH-positive axons morphology [60]. Despite these differences that may underlie behavioral abnormalities, direct intranasal administration of dopamine hydrochloride revealed an essentially identical therapeutic profile in both ASD models. It restored social balance without reducing repetitive behaviors or negatively affecting locomotion or anxiety-like responses [60].

Another key monoamine, serotonin, is a critical modulator of neuronal activity and behavior [61]. A recent study by Rahdar and colleagues (2024) demonstrated that repeated administration of the 5-HT_7_ receptor agonist (LP-211) can significantly reduce stereotypical behaviors and improve motor coordination and recognition of new objects in VPA-exposed rats [62]. However, the effects of LP-211 on social deficits, the core ASD symptom, were not assessed in this study.

To elucidate the mechanisms underlying the beneficial effect of memantine or aripiprazole administered separately or in combination on ASD-like symptoms, Zohny and colleagues [51] analyzed hippocampal tissue from VPA-exposed rats after treatment [51]. The pharmacological interventions restored the GABA/glutamate balance by increasing GABA levels while decreasing glutamate concentrations. Additionally, all treatment regimens increased the mRNA level of *Glt-1* and the expression of BDNF. Furthermore, immunofluorescence studies showed that chronic administration of the drugs tested also decreased levels of GFAP, Caspase-3, and BAX in the CA1 and DG of the hippocampus. The synergistic effect of memantine and aripiprazole increased *p*-CREB levels [51]. In another glutamatergic-targeted study, NitroSynapsin administration in *Mef2c*^*+/−*^ mice increased the number of NeuN-positive cells and the levels of VGAT and parvalbumin-positive synapses, without altering the number of parvalbumin-positive neurons within the hippocampus. Additionally, NitroSynapsin reduced the level of VGLUT2, the VGLUT1/VGAT ratio, and VGLUT2/VGAT ratio, as well as the number of hippocampal GFAP-positive cells [52]. In turn, in the VPA-induced rodent model of ASD, 4-week treatment with risperidone at both tested doses (1 and 3 mg/kg) increased *Adar2* relative gene expression and decreased GluA2 Q:R ratio in the hippocampus and the PFC. Risperidone also significantly increased the number of viable neurons and BCL2-positive cells, reducing the number of caspase-3-positive cells and apoptotic processes. Furthermore, risperidone alleviated hippocampal and cortical oxidative and nitrosative stress, normalized elevated cytochrome-c, LDH, caspase-3, MDA, and NO levels while increasing decreased TAC and GSH [53].

In addition to comprehensive behavioral analyses, Derieux et al. [55] investigated transcriptomic alterations following NaBr treatment in *Oprm1*-KO mice across five brain regions (NAc, CPu, VP/Tu, MeA, and VTA/SNc) [55]. At the transcriptional level, chronic NaBr increased the expression of genes coding for chloride ion transporters, GABA-A receptor subunits, oxytocin, and mGlu4 receptor within the reward/social circuit. However, the observed mRNA levels of the genes selected for study correlated poorly with social interaction parameters [55].

In *Fmr1*-KO mice, in which pharmacological intervention with PD158780 (ErbB inhibitor) led to a reduction in repetitive behaviors, electrophysiological experiments showed that the behavioral effects were due to a reduction in mGluR1/5-activated currents (I_DHPG_) and the co-occurrence of spontaneous firing hyperactivity and hyperexcitability of SNpc DA neurons [57]. In the same ASD model, administration of dopamine hydrochloride decreased TH expression in the striatum. In contrast, in BTBR mice, the same intervention, despite similar efficacy in treating social deficits, induced an opposite effect [58, 60]. When α5GABA-A receptor PAM, MP-III-022 was used in rats in the VPA model, an increase in mRNA level of *KCC2* was observed only in females treated with a dose of 1 mg/kg, while the mRNA level of *Gabra5* and *Nkcc1* remained unchanged across doses and animal sexes [58].

### Gut microbiota-based interventions

Gut microbiota-based therapeutic strategies represent a novel approach to the treatment of neuropsychiatric disorders [63–65]. The mechanisms behind the microbiota-gut-brain axis are still emerging and gut microbiota-based interventions were used in six studies. These studies utilized classical probiotics (*Lactobacillus and Bifidobacteria*) and FMT illustrating the effect of a targeted and more broad approach to targeting the gut microbiota. [66, 67] both used single bacterial strains (*Bifidobacterium longum* BB536 and *Bifidobacterium longum* CCFM1077 respectively) and reported minor behavioral improvements, although they were not consistent or extensive. The use of single bacterial strain to rescue behavioral deficits associated with ASD therefore remains uncertain. Similar findings were obtained when using *Lactobacillus spp., Bifidobacterium spp*. mixture with improvements restricted to sociability with no effect on grooming frequency or level of activity [68]. A more promising mild intervention is the use of combined prebiotic and probiotic mixtures to achieve additional synbiotic effect. Study of [69] which utilized *Lactobacillus reuteri C501* and inulin showed improvements in sociability, social novelty, and self-grooming time. These behavioral domains correspond to core diagnostic features of ASD, which include deficits in social behavior and increase in repetitive stereotypic behaviors that are the main diagnostic criteria [1]. While the effect of less specific interventions, such as FMT have a broader yet still positive effect on behavior. [70, 71] both reported improvements in social behavior and anxiety despite differences in FMT protocol.

Microbiota-based interventions have been shown to induce behavioral changes in ASD animal models, with metabolic modulation emerging as a primary pathway through which the gut microbiota exerts its influence on the CNS. Both a single bacteria [67, 69] and FMT interventions are capable of inducing extensive metabolic changes in the gut. Administration of *Bifidobacterium longum* CCFM1077 increased levels of tryptophan, reduced kynurenine, and changes in kynurenic acid/kynurenine ratio in the gut indicating modulation of tryptophan metabolism (Table 3) [67]. Gut metabolome changes translated to systemic circulation with lower levels of serum kynurenine, kynurenine/tryptophan ratio, and kynurenic acid/kynurenine ratio. While supplementation with *L. reuteri* C501 and inulin increased the concentration of SCFAs in feces and serum showing the metabolic effect extend beyond the gut [69]. FMT induced widespread cecal metabolome changes with 123 differentially expressed metabolites across multiple pathways [71]. Among microbiota-based interventions, FMT exerts the most extensive metabolic effects, but the precise outcome heavily relies on the donor’s microbial profile.

**Table 3:** Summary of global physiological, metabolic, and gut-microbiota changes after pharmacological and microbiota-based interventions in rodent models of ASD.

Reference	Animal model	Intervention	Blood markers	Physiological parameters	Global gene expression changes	Gut microbiota related changes
***Oxytocin signaling***						
**Bao et al**., [44]	Prenatal valproic acid (VPA)-induced, Wistar rats	Arginine vasopressin (*intranasal*, 400 µg/kg, 22 d)	Not assessed	↑ interleukin 4 production, ↑ brown fat differentiation	Not assessed	Not assessed
**Dai et al**., [31]	Prenatal valproic acid (VPA)-induced, Wistar rats	Oxytocin (*intranasal*, 20 µg; s.c., 3 µg, 7 d)	Not assessed	Not assessed	Not assessed	Not assessed
**Hörnberg et al**., [37]	*Nlgn3*-KO mice; *Fmr1*-KO mice	ETC-168 (*p.o*., 5 mg/kg, acute or 8-11 d); L-368,899 (oxytocin receptor antagonist, *i.p*., 10 mg/kg)	Not assessed	Not assessed	Not assessed	Not assessed
**Lindenmaier et al**., [40]	*16p11.2*^+/−^ mice; *Fmr1*-KO mice; *Shank3*-KO mice	Oxytocin (*intranasal*, 0.15 μg/10 μL, 28 d)	Not assessed	Not assessed	Not assessed	Not assessed
**Liu et al**., [43]	Prenatal valproic acid (VPA)-induced, Sprague Dawley rats	Atosiban (oxytocin receptor antagonist, *intranasal*, 100 μg/kg, 14 d)	Not assessed	Not assessed	Not assessed	Not assessed
**Moy et al**., **[**39**]**	BALB/cByJ mice	Oxytocin, (*i.p*., 1 or 2 mg/kg, acute or 4 d); TC-OT-39 (oxytocin analog, *i.p*., 30 or 50 mg/kg, acute or 4 d); [pGlu4,Cyt6]OT(4–9) (*i.p*., 1 or 2 mg/kg); [Cyt6] OT(5–9) (*i.p*., 1 or 2 mg/kg); Carbetocin (*i.p*., 3, 6, 10, 15, 20 mg/kg)	Not assessed	Not assessed	Not assessed	Not assessed
**Nagano et al**., [34]	*15q dup* mice	Oxytocin (*s.c*., 0.2-0.26 mg/kg, 15 d); 8OH-DPAT (5-HT_1A_ receptor agonist; *s.c*., 0.5 mg/kg, 15 d)	*DPAT* *↑* plasma oxytocin	Not assessed	Not assessed	Not assessed
**Pantouli et al**., [42]	*Oprm1-*KO mice	Oxytocin (*intranasal*, 0.15 IU ∼400 μg/kg, 0.3 IU ∼800 μg/kg or 0.6 IU ∼1600 μg/kg, acute or 17 d); LI183 (oxytocin receptor antagonist, *i.p*., 7.5 or 15 mg/kg)	Not assessed	Not assessed	Not assessed	Not assessed
**Sala et al**., [33]	*Oxtr*-KO mice	Oxytocin (*i.c.v*., 0.5 ng); Vasopressin (*i.c.v*., 0.5 ng); SR49059 (V1a receptor antagonist, (*i.c.v*., 0.5 ng)	Not assessed	Not assessed	Not assessed	Not assessed
**Shariatpanahi et al**., [41]	Prenatal valproic acid (VPA)-induced, Wistar rats	Oxytocin (*intranasal*, 1 μg/μL, 10 μL per nostril)	Not assessed	Not assessed	Not assessed	Not assessed
**Teng et al**., [38]	BALB/cByJ mice; C58/J mice	Oxytocin (*i.p*., 1 mg/kg, 1 d or 4 d)	Not assessed	Not assessed	Not assessed	Not assessed
***Neurotrasmission (GABA, glutamete, DA, 5-HT)***						
**Chao et al**., [60]	BTBR mice; *Fmr1*-KO mice	Dopamine hydrochloride (*intranasal*, 3 mg/kg)	*BTBR mice* Not assessed	*BTBR mice* Not assessed	*BTBR mice* Not assessed	*BTBR mice* Not assessed
			*Fmr1*-KO *mice* Not assessed	*Fmr1*-KO *mice* Not assessed	*Fmr1*-KO *mice* Not assessed	*Fmr1*-KO *mice* Not assessed
**D’Addario et al**., [57]	*Fmr1*-KO mice	PD158780 (ErbB inhibitor, *i.c.v*. into SNpc, 10 µM or *i.p*., 10 mg/kg)	Not assessed	Not assessed	Not assessed	Not assessed
**Derieux et al**., [55]	*Oprm1-*KO mice*;*	NaBr (*i.p*., 10, 30, 70, 125, 250 or 500 mg/kg, 18 d); NaBr (*i.p*., 250 mg/kg, 15 d); NaBr (*p.o*., 250 mg/kg, 5 d); KBr (*i.p*., 145 mg/kg, 18 d); Bumetanide (*i.p*., 0.5, 2 mg/kg, 18 d); VU0155041 (PAM of mGlu4 receptor, *i.p*., 1 mg/kg, 18 d); NaBr (*i.p*., 70, mg/kg, 18 d) + VU0155041 (*i.p*., 1 mg/kg, 18 d)	*Oprm1-*KO mice Not assessed	*Oprm1-*KO mice Not assessed	*Oprm1-*KO mice Not assessed	*Oprm1-*KO mice Not assessed
	*Fmr1*-KO mice;		*Fmr1-KO* mice Not assessed	*Fmr1-KO* mice Not assessed	*Fmr1-KO* mice Not assessed	*Fmr1-KO* mice Not assessed
	*Shank3*^*Δex13-16−/−*^ mice		*Shank3*^*Δex13-16−/−*^ mice Not assessed	*Shank3*^Δ*ex13-16−/−*^ mice Not assessed	*Shank3*^Δ*ex13-16−/−*^ mice Not assessed	*Shank3*^Δ*ex13-16−/−*^ mice Not assessed
**Dobrovolsky et al**., [50]	Valproic acid (VPA)-induced, Wistar rats (exposure pups)	Xenon (25% inhalation, 10 min)	Not assessed	Not assessed	Not assessed	Not assessed
**Habib et al**., [53]	Prenatal valproic acid (VPA)-induced, Wistar rats	Risperidone (*i.p*., 1 or 3 mg/kg, 28 d)	Not assessed	↑ body weight *(in both doses)*	Not assessed	Not assessed
**Rahdar et al**., [62]	Prenatal valproic acid (VPA)-induced, Wistar rats	LP-211 (5-HT_7_ receptor agonist, *i.p*., 1 mg/kg, 10 d)	Not assessed	**↔** body weight	Not assessed	Not assessed
**Santrač et al**., [58]	Prenatal valproic acid (VPA)-induced, Wistar rats	MP-III-022 (α5GABAAR PAM, *i.p*., 0.33 or 1 mg/kg, 7 d)	Not assessed	Not assessed	Not assessed	Not assessed
**Tu et al**., [52]	*Mef2c*^*+/−*^ mice	NitroSynapsin (*i.p*., 4.6 μmol/kg, twice a day for 90 d)	Not assessed	Not assessed	Not assessed	Not assessed
**Vicidomini et al**., [54]	*Shank3*^*Δ11 -/-*^ mice	CDPPB (mGlu5 receptor PAM, *i.p*., 3 mg/kg, acute/chronic)	Not assessed	Not assessed	Not assessed	Not assessed
**Yoshimura et al**., [56]	BTBR mice	GRN-529 (*i.p*., 3 mg/kg); 2-261 (*i.p*., 0.1, 0.3, 1, 3 mg/kg); 2-301 (*i.p*., 0.3, 1, 3 mg/kg); 2-313 (*i.p*., 1, 3 mg/kg); AVL-3288 (*i.p*., 0.3, 1, 3 mg/kg); 4-327 (*i.p*., 1, 3 mg/kg)	Not assessed	Not assessed	Not assessed	Not assessed
**Zohny et al**., [51]	Prenatal valproic acid (VPA)-induced, Wistar rats	Memantine (*i.p*., 20 mg/kg, 38 d); Aripiprazole (*i.p*., 3 mg/kg, 38 d); Memantine (*i.p*., 20 mg/kg, 38 d) + Aripiprazole (*i.p*., 3 mg/kg, 38 d)	*Memantine (i.p., 20 mg/kg, 38 d);* **↔** Body weight 4-6 weeks after treatment *Aripiprazole (i.p., 3 mg/kg, 38 d);* ↑ Body weight 4-6 weeks after treatment *Memantine (i.p., 20 mg/kg, 38 d) + Aripiprazole (i.p., 3 mg/kg, 38 d)* ↑Body weight 4-6 weeks after treatment	*Memantine (i.p., 20 mg/kg, 38 d);* Not assessed *Aripiprazole (i.p., 3 mg/kg, 38 d);* Not assessed *Memantine (i.p., 20 mg/kg, 38 d) + Aripiprazole (i.p., 3 mg/kg, 38 d)* Not assessed	*Memantine (i.p., 20 mg/kg, 38 d);* Not assessed *Aripiprazole (i.p., 3 mg/kg, 38 d);* Not assessed *Memantine (i.p., 20 mg/kg, 38 d) + Aripiprazole (i.p., 3 mg/kg, 38 d)* Not assessed	*Memantine (i.p., 20 mg/kg, 38 d);* Not assessed *Aripiprazole (i.p., 3 mg/kg, 38 d);* Not assessed *Memantine (i.p., 20 mg/kg, 38 d) + Aripiprazole (i.p., 3 mg/kg, 38 d)* Not assessed
***Anti-inflammatory***						
**Abdel-Haq et al**., [95]	*Shank3*^*Δ4-22*^ mice	7‑NI (nNOS inhibitor; *s.c*., PSARA gel formulation, 80 mg/kg)	initial ↑ 3-nitrotyrosine, normalization in day 5 after administration	Not assessed	Not assessed	Not assessed
**Cristiano et al**., [93]	BTBR mice; Prenatal valproic acid (VPA)-induced, B6 mice	MR‑39 (FPR2 agonist; *i.p*., 10 mg/kg, 8 d)	Not assessed	Not assessed	Not assessed	Not assessed
**Erdoğan et al**., [96]	Propionic acid (PPA)-induced, male Wistar rats	Pentoxifylline (*p.o*., 300 mg/kg, 15 d)	Not assessed	Not assessed	Not assessed	Not assessed
**Hidema et al**., [92]	*Oxtr*-KO mice; Prenatal valproic acid (VPA)-induced, C57BL6/J mice	Resveratrol (*i.p*., 30 mg/kg)	*Oxtr*-KO mice Not assessed	*Oxtr*-KO mice Not assessed	*Oxtr*-KO mice Not assessed	*Oxtr*-KO mice Not assessed
			*Prenatal valproic acid (VPA)-induced, C57BL6/J mice* Not assessed	*Prenatal valproic acid (VPA)-induced, C57BL6/J mice* Not assessed	*Prenatal valproic acid (VPA)-induced, C57BL6/J mice* Not assessed	*Prenatal valproic acid (VPA)-induced, C57BL6/J mice* Not assessed
**Özkul et al**., [94]	Propionic acid (PPA)-induced, male Wistar rats	Vardenafil (*p.o*., 3 mg/kg, 15 d)	Not assessed	Not assessed	Not assessed	Not assessed
**Zhang et al**., [97]	Prenatal valproic acid (VPA)-induced, Sprague Dawley rats	Aspirin (*i.p*., 1 mg/kg, 30 d)	Not assessed	Not assessed	Not assessed	Not assessed
***Microbiota***						
**Abuaish et al**., [70] **and** **Abujamel et al**., [66]	Propionic acid (PPA)-induced, male Sprague Dawley rats	Fecal microbiota transplant (*p.o*., 1 g/kg/day, 22 d); *Bifidobacterium longum* BB536 (*p.o*., 1 × 10⁹ CFU/day, 2 d); Fecal microbiota transplant (*p.o*., 1 g/kg/day, 30 d); *Bifidobacterium longum* BB536 (*p.o*., 1 × 10⁹ CFU/day, 30 d)	Not assessed	Not assessed	Not assessed	*FMT* ↓ abundance of *C. perfringens*, ↑ abundance of *C. cluster IV*, **↔** alpha- or beta- diversity, ↑ abundance of *Paraeggerthella, Dorea, Butyricicoccus, Lachnospiraceae, Clostridiales* *Bifidobacterium* ↓ abundance of *C. perfringens, ↑ abundance of C. cluster IV*, **↔** alpha- or beta- diversity, ↑ abundance of *Marvinbryantia, Dorea, Lactobacillaceae*
**Kong et al**., [67]	Prenatal valproic acid (VPA)-induced, Wistar rats	*Bifidobacterium longum* CCFM1077 (*p.o*., 10⁹ CFU/mL); Risperidone (*p.o*., 1.6 g/100 g)	*Bifidobacterium longum* CCFM1077 No changes in serum tryptophan ↓ Plasma kynurenine ↓ Plasma kynurenine/tryptophan ratio ↑ Plasma kynurenic acid/ kynurenine ratio ratio	Not assessed	Not assessed	*Bifidobacterium longum* CCFM1077 ↑ gut tryptophan, ↓ gut kynurenine, ↑ gut kynurenic acid/ kynurenine ratio, **↔** alpha diversity, Changes in beta diversity, *↑ Ruminococcaceae NK4A214* group, ↑ *Lachnoclostridium*, ↓ *Ruminococcus* *UCG-010*, ↓ *Intestinimonas*, ↓ *Rodentibacter*, ↓ *Marvinbryantia*
**Mintál et al**., [68]	Prenatal valproic acid (VPA)-induced, Wistar rats	Probiotic mixture (*Lactobacillus spp., Bifidobacterium spp*; *p.o*., 14 d)	Not assessed	**↔** body weight, food and water consumption	Not assessed	**↔** SCFAs in feces
**Wang et al**., [71]	Prenatal valproic acid (VPA)-induced, C57BL6/J mice	Fecal microbiota transplantation from healthy patients (*p.o*., 21 d)	123 differentially expressed metabolites in multiple subclasses. TD_FMT group exhibited enriched in specific metabolic pathways, including L-glutamic acid, (S)-glutamic acid, glutathione, oxidized L-proline, and L-asparagine.	Not assessed	Changes in colon metabolic pathway, glycosphingolipid biosynthesis, alpha-linolenic acid metabolism, serotonergic synapse pathway and glutamatergic metabolism DEGs after FMT	Differences in alpha- and beta- diversities after FMT. Distinct gut microbiota composition in ASD_FMT and TD_FMT Changes in phylum level. *↑Bacteroides* and *Odoribacter* in ASD_FMT *↑Akkermansia and Erysipelatoclostridium* in TD_FMT Relative abundance of *Turicibacter* and *Alistipes* positively correlated with the improvement in behavior
**Wang et al**., [69]	Prenatal valproic acid (VPA)-induced, C57BL6/J mice	*Lactobacillus reuteri C501* (*p.o*., 10^8^ CFU, 28 d); Inulin (*p.o*., 1% w/v in drinking water, 28 d); Synbiotic (*p.o*., *L. reuteri C501*, 10^8^ CFU + inulin, 1% w/v, 28 d)	Not assessed	**↔** body weight	Not assessed	Synbiotic effects: *↑ L. reuteri* levels in feces PLS-DA diversity differences induced by synbiotic administration, *↑ Allobaculum*, *Bifidobacterium*, and *Parasutterella*, ↓ *Odoribacter* and *Roseburia*, Changes in metabolic profile of serum, *↑* SCFAs levels in feces and serum, *↑* GHB, 3-hydroxybutyric acid, *N*-acetyltryptophan, malonic acid, acetic acid, ↓ hippuric acid levels in serum
***Cannabinoids***						
**Loomis et al**., [85]	Prenatal valproic acid (VPA)-induced, Wistar rats	JZP541 (*i.p*., acute 100 mg/kg or 10, 30, 100 mg/kg, 5 d); Risperidone (*i.p*., 0.125 mg/kg, 5 d)	Not assessed	Not assessed	Not assessed	Not assessed
**Pedrazzi et al**., [83]	Prenatal valproic acid (VPA)-induced, Swiss mice	Cannabidiol (*i.p*., 30 or 60 mg/kg)	Not assessed	Not assessed	Not assessed	Not assessed
**Poleg et al**., [82]	*InsG3680 Shank3* mice	Avidekel oil (*p.o*., 5 ml/kg, (CBD:THC ratio 20:1, 25 mg/kg CBD, 1 mg/kg THC, twice a week for 21 d); 5 ml/kg CBD oil (*p.o*., 25 mg/kg CBD twice a week for 21 d); Erez oil (*p.o*., 1 mg/kg THC, no CBD twice a week for 21 d); THC oil (*p.o*., 1 mg/kg THC twice a week for 21 d); AM-251 (CB1 receptor antagonist, *i.p*., 3 mg/kg); WIN55,212-2 (CB1 receptor agonist, *i.p*., 0.5 mg/kg)	*ADKL:* *↑ s*erum CBD and THC, ↓ serum 2-AG and anandamide/AEA *Erez:* *↑ s*erum THC, ↓ serum 2-AG, ↓ serum AEA	**↔** body weight	Not assessed	Not assessed
**Zamberletti et al**., [84]	Prenatal valproic acid (VPA)-induced, Sprague Dawley rats	Cannabidivarin (*i.p*., 0.2, 2, 20, 100 mg/kg, 23 d - symptomatic schedule); Cannabidivarin (*i.p*., 0.2, and 20 mg/kg, 12 d - preventive schedule)	Not assessed	Not assessed	Not assessed	Not assessed
***Metabolic***						
**Elnahas et al**., [75]	Prenatal valproic acid (VPA)-induced, Wistar rats	Metformin (*i.p*., 100 mg/kg, 38 d); Risperidone (*i.p*., 1 mg/kg, 38 d); Metformin (*i.p*., 100 mg/kg, 38 d) + Risperidone (*i.p*., 1 mg/kg, 38 d)	Not assessed	*Risperidone:* restored body weight to WT levels *Metformin:* **↔** body weight *Metformin+Risperidone:* **↔** body weight	Not assessed	Not assessed
**Mirza & Sharma**, [76]	Prenatal valproic acid (VPA)-induced, Wistar rats	Pioglitazone (*p.o*., 10 or 20 mg/kg, 28 d)	Not assessed	Not assessed	Not assessed	Not assessed
**Sandhu et al**., [77]	Prenatal valproic acid (VPA)-induced, Wistar rats	Pioglitazone (*s.c*., 2.5, 5, 10 mg/kg, 30 d)	Not assessed	Not assessed	Not assessed	Not assessed
**Wang et al**., [74]	BTBR mice	Metformin (*i.p*., 200 mg/kg, 8 d)	Not assessed	**↔** body weight	Not assessed	Not assessed
***Purines***						
**Hirsch et al**., [88]	Prenatal valproic acid (VPA)-induced, Wistar rats	Suramin (purinergic antagonist, *i.p*., 20 mg/kg)	Not assessed	Not assessed	Not assessed	Not assessed
**Naviaux et al**., [86]	Maternal immune activation (MIA) - gestational poly(IC) exposure, C57BL/6J mice	Suramin (purinergic antagonist, *i.p*., 10 or 20 mg/kg; weekly 3x)	*↑* plasma immunoglobulins, *↑* plasma corticosterone *(20 mgkg)*	restoration of normal basal body temperature *(20 mgkg)*	Not assessed	Not assessed
**Naviaux et al**., [87]	Maternal immune activation (MIA) - gestational poly(IC) exposure, C57BL/6J mice	Suramin (purinergic antagonist, *i.p*., 20 mg/kg)	Not assessed	restored brain weight/weight ratio in both sexes	Not assessed	Not assessed
**Vitamins**						
**Du et al**., [89]	Prenatal valproic acid (VPA)-induced, Wistar rats	Vitamin D3 (*i.m*., 80,000 IU/kg)	↑ serum 25(OH)D3 levels	↑ body weight ↑ eye opening category score	Not assessed	Not assessed
**Luo et al**., [90]	Prenatal valproic acid (VPA)-induced, Sprague Dawley rats	Retinoic acid (*p.o*., 6 mg/kg, 21 d)	Not assessed	Not assessed	Not assessed	Not assessed
**Zhu et al**., [91]	Prenatal valproic acid (VPA)-induced, Sprague Dawley rats	Retinoic acid (*p.o*., 1 mg/kg, 10 d)	Not assessed	restored brain weight/weight ratio in both sexes	Not assessed	Not assessed
***Others (calcium, adrenergic receptor, histone modifications)***						
**Alhamami et al**., [101]	Prenatal valproic acid (VPA)-induced, Sprague Dawley rats	6-hydroxydopamine (6‑OHDA, *i.c.v*. into CV4; 75 µg/µl, 2 d) ± LPS (*i.p*., 500 µg/kg)	Not assessed	Not assessed	Not assessed	Not assessed
**Carreno-Muñoz et al**., [98]	*Fmr1*-KO mice	BMS-204352 (BKCa agonist, *i.p*., 2 mg/kg)	Not assessed	Not assessed	Not assessed	Not assessed
**Feng et al**., [100]	Prenatal valproic acid (VPA)-induced, Sprague Dawley rats	Calcium hexacyanoferrate (III) nanocatalysts (CaH NCs, *i.c.v*., 0.2 mg/kg)	Not assessed	**↔** body weight **↔** eye opening score ↑ plane correction	Not assessed	Not assessed
**Rapanelli et al**., [99]	*Shank3*^*+/ΔC*^ mice; *Shank3*^*E13*^ mice; *Cul3*^*f/−*^ mice	GSK-LSD1 (LSD1 inhibitor, *i.p*., 5 mg/kg, 3 d); ORY-1001 (LSD1 inhibitor, *i.p*., 0.015 mg/kg, 3 d); AAV2-CMV-EGR1-Flag (Egr1 AAV into PFC, *i.c.v*., 0.3 μl per hemisphere)	*Shank3*^*+/ΔC*^ mice Not assessed	*Shank3*^*+/ΔC*^ mice Not assessed	*Shank3*^*+/ΔC*^ mice Not assessed	*Shank3*^*+/ΔC*^ mice Not assessed
			*Shank3*^*E13*^ mice Not assessed	*Shank3*^*E13*^ mice Not assessed	*Shank3*^*E13*^ mice Not assessed	*Shank3*^*E13*^ mice Not assessed
			*Cul3*^*f/−*^ mice Not assessed	*Cul3*^*f/−*^ mice Not assessed	*Cul3*^*f/−*^ mice Not assessed	*Cul3*^*f/−*^ mice Not assessed

↑ significant increase, ↓ significant decrease/reduction, ↔ no significant change, *AEA* anandamide, *CFU* colony-forming units, *d*, days *icv* intracerebroventricular administration, *im* intramuscular administration, *ip* intraperitoneal administration, *po* oral administration, *PAM* positive allosteric modulator, *PFC* prefrontal cortex, *SCFAs* short chain fatty acids, *SNpc* substantia nigra pars compacta, *THC* tetrahydrocannabinol, *2-AG* 2-arachidonoylglycerol.

### Targeting metabolism

ASD is associated with high levels of metabolic disturbance with as many as 30% of children with ASD experiencing metabolic abnormalities, such as differences in amino acid metabolism [72]. Additionally, drugs frequently employed in the modulation of behavioral symptoms contribute to the development of metabolic syndrome with most common metabolic symptom being dyslipidemia [73]. Metabolic modulators such as metformin and pioglitazone are receiving more attention in the context of ASD, not only as supplementary therapies aimed at reducing the metabolic side effects of conventional medication, but also as potential primary interventions. Metformin treatment was successful in improving sociability in three-chamber test and the frequency of repetitive behaviors in marble burying and self-grooming tests in prenatal VPA exposure and BTBR mouse models [74, 75]. Similar results on social behavior were observed with the use of pioglitazone, which is an anti-diabetic drug and is frequently prescribed together with metformin. Both studies [76, 77] reported increased sociability and social novelty in the three-chamber test when using 10 mg/kg of pioglitazone in the VPA mouse model. Although the effect on repetitive behaviors remains inconclusive with [76] reporting an increase in self-grooming, while [77] reported a reduced number of buried marbles even at the lower (5 mg/kg) dose.

These behavioral outcomes are most likely facilitated through a reduction of oxidative stress and neuroinflammation. Studies utilizing metformin reported lower levels of TNF-α, S6K, IL-1β, and mTOR in the PFC [74, 75]. Inflammation signaling pathways are frequently dysregulated in ASD animal models and may contribute to the behavioral symptoms [78]. Administration of pioglitazone reduced levels of IL-6, TNF-α and increased levels of IL-10 [76]. Similar findings were observed in the brainstem and cerebellum indicating that these effects are not region-specific but rather reflect a more brain-wide neuroinflammatory and antioxidant modulation. The anti-inflammatory effect of pioglitazone on the brain is further supported by increased levels of glutathione and catalase in the PFC and cerebellum [77]. Metformin interventions had similar anti-inflammatory effects to a lesser extent and were comparable to risperidone [74, 75]. Combined therapy of metformin and risperidone provided a synergistic effect on reducing TNF-α and IL-1ß in the PFC and hippocampus [75]. Additionally, these effects are achieved without any impact on body weight [74, 75]. These findings indicate the potential of metformin and pioglitazone in alleviating neuroinflammation and in supporting their use with currently used medications.

### Cannabinoids for modulation of ASD symptoms

The endocannabinoid system (ECS) is a multimodal neuromodulatory network that modulates social behavior and reward-seeking behaviors in general [79–81]. Various natural cannabinoids (cannabidiol (CBD), tetrahydrocannabinol (THC), cannabidivarin (CBDV), Avidekel oil, Erez oil) and their preparations are actively researched to alleviate social deficits in ASD. THC rich Erez oil increased sociability and reduced the number of buried marbles without any effect on activity [82]. While THC alone only improved social behavior. The effects of CBD on social behavior are heavily influenced by the administered concentration. Lower concentrations (25 mg/kg [82], 30 mg/kg [83]) of CBD had no effect on sociability. CBD rich Avidekel oil (extracted from Avidekel cannabis flower) induced lower sociability, reduced grooming time and shorter distance traveled, indicating an overall inhibitory effect [82]. While 60 mg/kg increased sociability, reduced repetitive behaviors and improved memory [83]. Similarly, CBDV increased sociability and memory, while reducing self-grooming and activity [84]. Naturally occurring non-psychoactive cannabinoids, such as CBD and CBDV show promise in alleviating behavioral deficits. However, the detected reduction in distance traveled by both compounds limits the significance of reduced self-grooming and introduces limitations to clinical application [82–84]. Novel cannabinoid formulations, such as JZP541 (a botanical mixture containing various cannabinoids with a small amount of THC) increased sociability and reduced repetitive behaviors in a wide range of concentrations (10 – 100 mg/kg) suggesting the involvement of the ECS in alleviating ASD-like symptoms. Although the 100 mg/kg dose lowered time in the center in the open field test, indicating increased anxiety [85].

Administration of JZP541 induced a dose-dependent response in gene expression within the frontal cortex, with the 10 mg/kg dose altering the expression of only seven genes, whereas the 100 mg/kg dose affected more than 1300 genes. Only a small subset of these genes was associated with ASD, while the large number of affected genes suggests a broad modulatory impact on the CNS, particularly on genes involved in synaptic function and neurotransmission [85]. Similarly, administration of Avidekel oil produced comparable transcriptional changes with pronounced changes in action potential and nerve impulse transmission pathways in the cerebellum supporting the idea of general neuromodulatory effects of the cannabinoid-based compounds [82]. Although CBDV treatment did not induce transcriptional changes in the PFC, it altered microglial cell morphology and reduced TNF-α expression, indicating a degree of anti-inflammatory modulation. Additionally, reduced expression of CB1 receptor and monoacylglycerol lipase indicates a direct regulation of the ECS through CB1-receptor-depended signaling [84]. Together, these findings suggest that cannabinoid-based interventions exert broad neuromodulatory and anti-inflammatory effects, acting through both transcriptional and endocannabinoid signaling pathways to influence neuronal excitability and synaptic function.

### Purine-based intervention

All reviewed studies [86–88] reveal a consistent pattern of findings. The studies, which include maternal immune activation (MIA) in mice (poly (I:C)) and prenatal VPA exposure in rats, demonstrate that antipurinergic therapy (APT) with suramin (20 mg/kg) results in improvements of impaired social behaviors. In both models, suramin administration enhanced sociability, increased the time spent in the social chamber, and increased sociability and social sniffing during the three-chamber test. Furthermore, Hirsch et al. [88] observed that the administration of suramin did not exert an influence on the time and number of instances of self-grooming in the open field test [88]. The study also revealed that the administration of sumarin did not affect the total time and number of interactions, nose-to-nose sniffing, anogenital inspection, and flank exploration. However, suramin increased the number of following behaviors in the reciprocal social interaction test. Improvements were also observed in elevated plus maze and open field tests, as indicated by increased time spent in the open arm or in the central square. In the MIA model, the administration of suramin resulted in an increased novelty preference in the T-maze test [86, 87]. Furthermore, improvements were observed in tasks assessing motor coordination in the rotarod test.

At the molecular level, suramin intervention resulted in substantial changes to the expression of genes and proteins involved in neurochemical and metabolic pathways associated with purinergic transmission and mitochondrial activity. In a 2020 study, Hirsch et al. observed that suramin did not induce significant alterations in the mRNA levels of purinergic receptors, as well as in the levels of cytokines and interleukins, in the PFC of rats exposed to VPA [88]. In addition, Naviaux et al. [86] discovered that suramin influenced the expression of numerous genes implicated in the function of the mitochondrial respiratory chain complexes I and IV [86]. The investigation further revealed an augmentation in the expression of purinergic receptors P2Y2 and P2X7, alongside an alteration in the ratio of pERK1/2/ERK1/2 and pCAMKII/CAMKII. Furthermore, histological analyses revealed an increased number of Purkinje cells in the cerebellum following treatment.

These converging behavioral and molecular results suggest that therapeutic effects of suramin drove coordinated molecular and behavioral improvements in various ASD models.

### Vitamin-based interventions

In all three reviewed studies [89–91] performed on rats, a prenatal VPA model was used and reported impairments in social behaviors. Intervention with retinoic acid (6 mg/kg for 21 days or 1 mg/kg for 10 days) significantly improved social functioning, as indicated by increased time spent in the social novelty chamber assessed by the three-chamber test. Additionally, changes observed in the open field test (e.g., increased time in the center) suggest a modulatory effect on anxiety-related behavior. Similarly, Du et al. reported that high-dose vitamin D₃ treatment (80,000 IU/kg) reduced self-grooming behavior and altered olfactory and social interaction patterns [89]. Concurrently, retinoic acid (6 mg/kg) administration reduced repetitive behaviors in the self-grooming test, as evidenced by a consistently observed decrease in self-grooming duration, a hallmark of stereotyped behavior [90].

At the molecular level, retinoic acid intervention resulted in substantial changes to the expression of genes and proteins that play a role in the function of microglia and the regulation of neuroinflammation in the PFC. This included increased protein  expression of ARG-1, RARα, and TREM2, and decreased expression of iNOS [90]. Furthermore, immunofluorescence analysis revealed a reduced number of IBA-1-positive cells and increased microglial branching complexity. Zhu et al. [91] found that retinoid treatment affected the expression of multiple genes related to synaptic transmission and neuroplasticity [91]. This included a reduction in mRNA levels of *Grin2b*, *Nrxn1*, *Cacna1e*, and *Gabrb2*, as well as the dysregulation of other key genes, such as *Reelin*, *Mecp2*, *Tbr1*, and *Kenmal*. These molecular changes were accompanied by altered functional brain connectivity, as indicated by BOLD-fMRI, with disrupted connectivity primarily observed in fronto-striatal and hypothalamic–hippocampal circuits.

Overall, the results indicate that vitamin D₃ and retinoic acid interventions have the potential to ameliorate core behavioral abnormalities. Molecular dysfunctions related to microglial activation and neurotransmission pathways appear to contribute to the behavioral phenotype in the VPA model. As a result, targeted nutritional interventions may offer a promising strategy for therapeutic modulation.

### Anti-inflammatory-based interventions

All studies reviewed regarding pharmacological interventions for anti-inflammatories in ASD models demonstrated significant changes in animal behavior. The findings of the studies in this section demonstrate an increased duration spent in the social chamber, sociability, or social novelty [92–97]. Abdel-Haq et al. [95] observed that treatment of Shank3^*Δ4-22*^ mice with 7-NI (nNOS inhibitor) resulted in an increase in time spent in the open arms of the elevated plus maze and an enhancement in cognitive behavior, as measured by the novel object recognition test [95]. Moreover, research utilising the prenatal VPA model has demonstrated that treatment with MR-39 (FPR2 agonist) enhances social interactions without eliciting alterations in other core ASD behaviors, such as marble burying and self-grooming, in BTBR mice [93]. Conversely, in the same model, aspirin administration has been shown to reduce stereotyped behaviors and anxiety-like responses [97]. Furthermore, research has demonstrated that propionic acid (PPA)-based models have shown indications of improved anxiety-like behaviors and exploratory and cognitive test results following intervention with pentoxifylline [96]. A similar outcome has been observed with Vardenafil treatment [94]. A thorough review of studies examining anti-inflammatory pharmacological interventions in animal models of ASD reveals a consistent pattern of beneficial behavioral effects.

Beyond behavioral improvements, studies have demonstrated that anti-inflammatory pharmacological interventions exert significant molecular effects in examined models, modulating key pathways involved in neuroinflammation, glial activation, and neuronal signaling, mostly in the hippocampus and cerebellum. As demonstrated in the study by Cristiano et al. [93], treatments such as MR-39 increased the mRNA levels of *Fpr2*, *Il-10* and *LX4A*, while reducing the mRNA levels of *Il-1β* and *Tnf*-*α* in the hippocampus of BTBR mice, and similarly in B6 mice treated with VPA [93]. In addition, the PPA model demonstrated that pentoxifylline intervention resulted in augmented neuronal density in CA1 and CA3 of the hippocampus, elevated Purkinje cell numbers, and diminished GFAP immunoreactivity and IL-17 and TNF-α levels in the brain [96]. This effect is analogous to that observed following Vardenafil intervention [94]. It is interesting to note that resveratrol has been observed to increase the mRNA levels of *Egr3* and *Sirt1* in the amygdala (Hidema et al., [92]), while aspirin treatment has been observed to increase the expression of p-ACC and p-AMPK in the hippocampus [97].

Interventions aimed at reducing inflammation in animal models of ASD have been shown to have a positive effect on behavior and cognition, whilst simultaneously reducing neuroinflammation and supporting the restoration of neuronal integrity in key brain regions.

### Other novel treatment options

In addition to the previously mentioned interventions, further analysis is warranted on studies that do not align with the previously delineated classifications. These studies may offer novel insights and significant results that are pertinent to the broader field. Carreno-Muñoz et al. [98] observed that treatment with BMS-204352 (a BKCa agonist) resulted in a reduction of repetitive and anxious behavior in *Fmr1*-KO mice [98]. In a similar study, Rapanelli et al. [99] observed increased levels of self-grooming in *Shank3*^*E13*^ and *Cul3*^*f/−*^ mice treated with GSK-LSD1 (a LSD1 inhibitor) or ORY-1001 (another LSD1 inhibitor) [99]. The researchers also noted an increased sociability index, with no changes in anxiety behavior. Furthermore, Feng et al. [100] demonstrated that the treatment with calcium hexacyanoferrate (III) nanocatalysts resulted in enhanced time spent in the social or social novelty chambers, as well as improved exploratory and cognitive behavior [100]. A single study, namely Alhamami et al. [101], yielded contradictory findings [101]. This study demonstrated that the administration of 6-hydroxydopamine led to an increase in self-grooming duration.

At the molecular level, 6-hydroxydopamine treatment has been observed to increase the levels of mRNA for *Il-6*, *Tnf-α*, and *NF-κ*, while decreasing the protein expression of GPX-1 and COX-2 in the PFC [101]. Treatment with calcium hexacyanoferrate (III) nanocatalysts in RNA-seq analysis has been shown to result in the identification of 280 differentially expressed genes in the hippocampus [100]. This treatment has been found to lead to a decrease in the levels of ROS, Iba-1, and GFAP immunoreactivity. In addition, GSK-LSD1 treatment (5 mg/kg, 3 days) resulted in alterations to 230 differentially expressed genes, including Egr1 and Egr4, in the PFC. Furthermore, it led to an increase in the level of H3K4me2 immunoreactivity in S*hank3*^*+/ΔC*^ mice [99].

Several novel interventions, including BKCa agonists, LSD1 inhibitors, and calcium hexacyanoferrate (III) nanocatalysts, have been observed to exert varied effects on repetitive, social, and cognitive behaviors in mouse models. These interventions have also been shown to induce corresponding molecular changes in gene expression and inflammatory markers. However, it should be noted that some findings remain contradictory.

## Risk of bias assessment

**Fig. 3:**
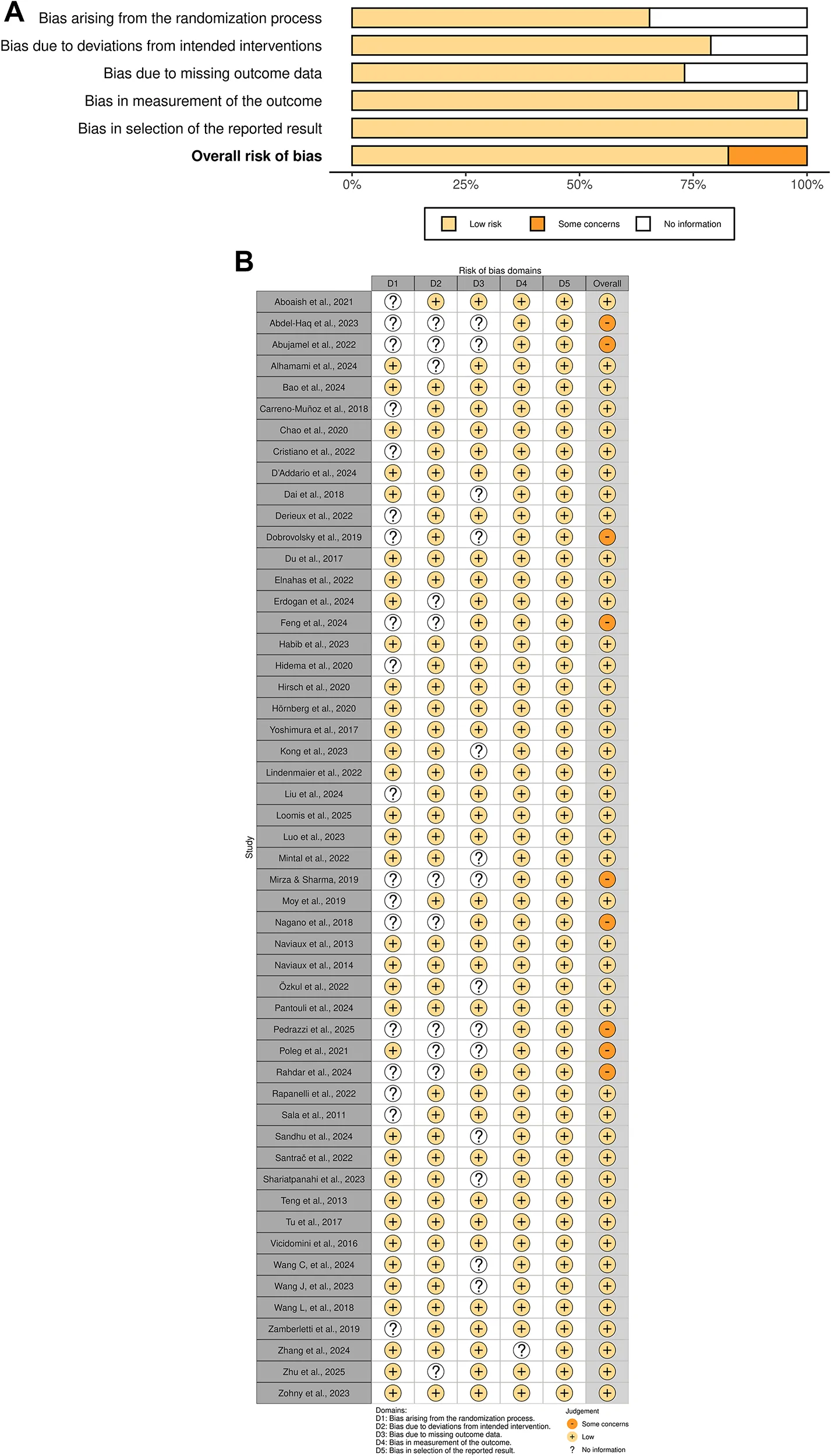
Risk of bias assesment using modified SYRCLE’s RoB criteria for animal studies. **A** Weighted bar plots of the distribution of risk-of-bias judgements within each bias domain; (**B**) “Traffic light” plots of the domain-level judgements for each individual result. Figure was produced using RoB visualization tool (robvis) [139].

The risk of bias assessment revealed low risk of bias in selected studies. A total of 21 studies exhibited no signs of bias, with an overall low risk (Fig. 3). More than half (31) of the studies showed at least some concerns, primarily due to insufficient methodological reporting or unclear information related to selection, performance, and detection biases. 10 studies exhibited signs of insufficient reporting in at least two categories. This pattern likely reflects broader flaws in the reporting standards of preclinical animal research rather than issues specific to the included publications. A summary table of risk of bias across the included studies was generated to visualize the distribution of potential biases and to inform the interpretation of results. Given the substantial behavioral variability characteristic of ASD, insufficient sample sizes represent an important source of bias in preclinical studies. It has been suggested that, when using rodent models of ASD, approximately 15–20 animals per genotype, treatment, and sex are required to ensure adequate power and reliability of behavioral outcomes [102]. Therefore, studies employing smaller experimental groups should be interpreted with caution and may require independent replication before firm conclusions can be drawn. Additionally, the authors acknowledge the general positive bias of the published results, the lack of replicative validation studies, and the resulting risk of overestimating treatment efficacy in preclinical models that would provide confidence for the current findings and improve translational significance.

## Discussion

**Fig. 4:**
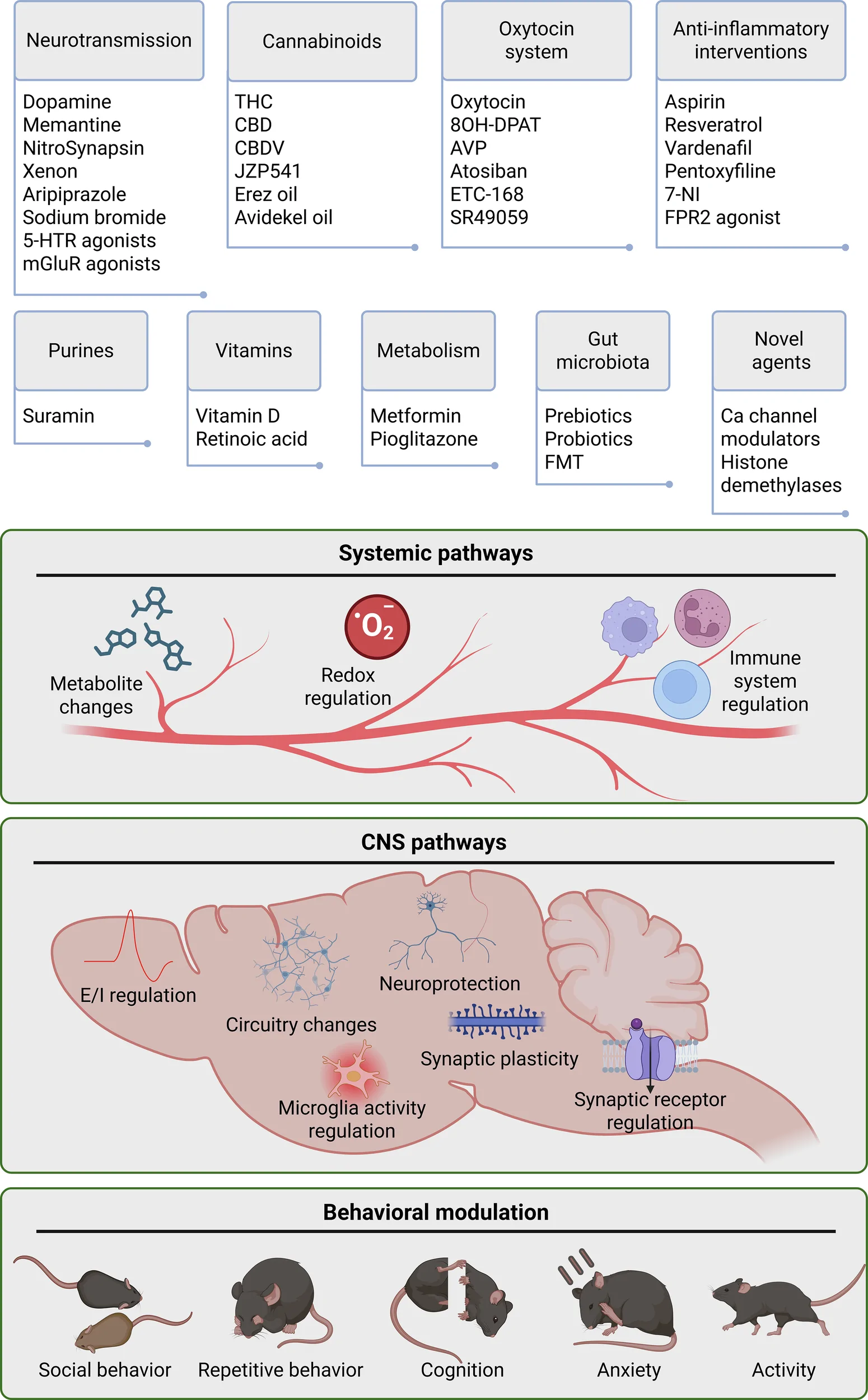
Overview of the investigated interventions, their primary targets in systemic circulation and the CNS with their associated behavioral outcomes. Created in BioRender.com.

This systematic review comprehensively evaluated the efficacy and underlying mechanisms of both pharmacological and microbiota-based therapeutic strategies in preclinical models of ASD. We specifically focused on behavioral outcomes, which highlights the functional significance of the findings. The reviewed studies have demonstrated that a variety of interventions can be effectively utilized to ameliorate typical ASD behavioral deficits. Despite heterogeneity in ASD animal models and experimental design, most studies reported significant improvement in at least one key ASD behavioral trait. Together, these findings highlight the diverse nature of ASD pathophysiology, in which inflammatory, metabolic, and synaptic mechanisms all contribute to the behavioral symptom presentation (Fig. 4). This mechanistic diversity strengthens the growing recognition that treatment strategies extending beyond the CNS, particularly those involving the gut microbiota or peripheral metabolic pathways, may offer complementary therapeutic benefits.

Among all the pharmacological and microbiota-based interventions reviewed, treatment with oxytocin has been the most extensively investigated. Eight studies have assessed its direct impact on the amelioration of core ASD symptoms. The rationale for this strategy lies in both the physiological profile of oxytocin and clinical observations indicating lower circulating oxytocin levels in children with ASD compared to neurotypical individuals. However, these differences diminish during adolescence and adulthood [103, 104]. This age-dependent variability in oxytocin signaling may delineate a developmental window in which such therapy could be most effective.

In the preclinical studies, exogenous oxytocin administration proved to be a promising therapeutic approach, effectively enhancing social behaviors while reducing repetitive behaviors in rats prenatally exposed to VPA [31, 41] and BALB/cByJ mice [39]. In other ASD models, such as *15q dup* mice [34], *Oprm1*-KO mice [42], *Oxtr*-KO mice [33], and C58/J mice [38], oxytocin selectively improved social deficits, without influencing other ASD symptoms. Notably, in models including *16p11.2*^*+/*-^, *Fmr1*-KO, and *Shank3*-KO mice, oxytocin failed to rescue ASD-like phenotypes [40]. An intriguing observation was reported in *Oprm1*-null mice, in which prior studies identified oxytocinergic dysfunction. Chronic intranasal oxytocin administration improved social behaviors, whereas the same regimen in wild-type mice induced pronounced and lasting social impairments [42, 105]. This suggests that a pre-existing deficit in the oxytocinergic system may be a prerequisite for beneficial outcomes of oxytocin treatment. Supporting this, VPA-exposed rats characterized by reduced cerebrospinal oxytocin levels and a decreased number of oxytocin-immunoreactive cells in the hypothalamus exhibited normalization of these parameters as well as rescue of the ASD-like phenotype following treatment [31].

Other important factors influencing oxytocin’s therapeutic efficacy may include the developmental stage, treatment schedule, and environmental context. Acute intranasal oxytocin improved social behavior in *Oprm1-*KO mice within 5 min after administration [42]. Nevertheless, acute exogenous oxytocin supplementation produced only a temporary prosocial effect [106, 107], likely due to short half-life of oxytocin in the brain [108, 109]. In contrast, treatment during the early postnatal period had a long-term benefit on sociability, which was observed in VPA-exposed rats during adolescence and in adult *15q dup* mice [31, 34]. These findings confirm that repeated oxytocin exposure during sensitive periods may upregulate endogenous oxytocinergic function and remodel social neural circuits [31, 38]. Furthermore, social-contextual administration enhanced and prolonged oxytocin’s prosocial effects in *Oprm1*-KO mice [42]. Sex differences represent another crucial aspect in the optimization of ASD therapies. While in male rats in the VPA model, exogenous oxytocin was effective in treating core ASD symptoms due to deficits in the oxytocin pathway [31], in females in the same model (characterized by increased hippocampal and cortical levels of oxytocin and OXTR), the OXTR antagonist Atosiban demonstrated therapeutic efficacy [43]. Liu et al. [43] linked this effect to restoration of synaptic plasticity impairments in females. These findings suggest sex-specific pathophysiology and the utility of oxytocin antagonists in selected subgroups [43].

The pharmacological limitations of oxytocin, including its short half-life and poor blood-brain barrier penetration, have prompted the search for more bioavailable synthetic analogues, as well as research into its active metabolites [38, 39]. Moy et al. [39] reported that two synthetic OXTR agonists, TC-OT-39 and carbetocin, despite showing activity in vitro, did not show prosocial effects in BALB/cByJ mice, although TC-OT-39 reduced marble-burying behavior. In contrast, the oxytocin metabolite OT(4–9) enhanced sociability in BALB/cByJ mice in a dose-dependent manner, with effects persisting up to 12 days after treatment, though without changes in repetitive behaviors [39]. This dissociation suggests that some prosocial effects of oxytocin may be mediated by downstream metabolites acting through noncanonical mechanisms beyond OXTR activation.

Beyond behavioral outcomes, mechanistic investigations have linked oxytocin treatment to modulation of neurotransmitter systems, including serotonin and dopamine, and to neuroprotective effects, such as reduced expression of the necroptosis markers RIP3 and MLKL in the hippocampus and amygdala of VPA-exposed rats [41]. These molecular alterations may underlie improvements in cognitive function and social memory. Examples include a study indicating functional interactions between oxytocin and serotonin systems in shaping social behavior. Supporting this interaction, in *15q dup* mice, postnatal activation of 5-HT_1A_ receptors normalized sociability in a manner blocked by OXTR antagonism, indicating an interaction between serotonin and oxytocin in the developmental programming of social circuits [34].

Despite mostly promising preclinical data, meta-analyses of clinical trials indicate that oxytocin treatment does not significantly improve impaired social and repetitive behaviors in adults with ASD [110], with only a moderate effect observed in children [111]. Collectively, these analyses indicate the need for more large-scale, rigorous, and multi-site randomized controlled trials to confirm the effectiveness of oxytocin as a treatment for ASD [110, 111]. Equally critical is the identification of biomarkers capable of defining ASD subgroups in whom intervention with oxytocin, a synthetic analog or even an antagonist of OXTR, might yield beneficial outcomes, given the heterogeneity in ASD etiology, phenotype, and treatment response [112, 113].

The therapeutic success of another large group of interventions often depends on correcting the underlying neurochemical disturbances observed in patients with ASD, in particular the excitation/inhibition (E/I) imbalance, and modulating key neurotransmitter systems [45, 114]. The efficacy of this approach has been demonstrated in studies showing improvements in social behaviors achieved by inhibiting excitatory activity through the use of NMDA receptor antagonists, e.g., subanesthetic doses of Xenon [50] and memantine in VPA-exposed rats [51] and NitroSynapsin in *Mef2c*^*+/−*^ mice [52], as well as through modulation of AMPA receptors by risperidone in a VPA model [53]. In addition to restoring glutamate/GABA balance, additional mechanisms may contribute to the observed behavioral effects. For example, therapy may work by enhancing CREB/BDNF signaling, increasing hippocampal mRNA level of *Glt-1*, thereby preventing neuronal apoptosis and reducing oxidative stress, as shown in the case of memantine treatment [51]. The results of a recent randomized clinical trial in which memantine was administered for 12 weeks confirmed the observations from preclinical studies, significantly improving social impairments in youths with ASD [115].

Beyond targeting ionotropic receptors, several studies have also reported the alleviation of core ASD symptoms through modulation of mGlu receptor activity, particularly mGlu4 and mGlu5 receptors [54–56]. Their involvement may result from their regulatory role in synaptic plasticity, which is impaired in numerous ASD models [116]. Similar to disturbances in glutamatergic signaling, preclinical and clinical data point to the primary inhibitory neurotransmitter of the CNS as important for a better understanding of the pathogenesis of ASD and for identifying new therapeutic strategies [117]. Thus, the use of PAMs of GABA-A receptors (MP-III-022 and 2–261) has been shown to alleviate symptoms characteristic of ASD in environmental and idiopathic models of ASD [53, 55].

In addition to modulating the two main neurotransmission pathways, other neurotransmitters, including dopamine, serotonin, and choline, should not be overlooked. The dopamine system, known for its role in reward, motivation, and memory, and serotonin, important in the regulation of anxiety and neurodevelopmental processes, have been implicated in ASD pathophysiology [118, 119]. Preclinical studies using intranasally administered dopamine have demonstrated therapeutic effects in two different mouse models of ASD - BTBR and *Fmr1*-KO mice [60]. Activation of serotonin receptor 7 (5-HT_7_R) using its agonist LP-211 showed beneficial effects on anxiety-like behaviors, social and cognitive deficits, in the VPA model [62]. However, a specific limitation of studies focusing on monoaminergic signaling is the omission of tests assessing aggressive behaviors (characteristic of patients with ASD) that appear to be modulated by dopamine and serotonin in individuals with ASD [59]. Furthermore, allosteric modulation of α7 nAChR with AVL-3288 alleviated social approach deficits and repetitive grooming behaviors in a BTBR mouse model [56]. The efficacy of compounds with such a broad spectrum of mechanisms of action confirms the complexity of ASD as a disorder [120]. It highlights the enormous challenges of developing targeted and safe and pharmacotherapy.

Gut-microbiota-based interventions represent a promising strategy for the treatment of neuropsychiatric disorders, including ASD. Although mechanistic understanding is only emerging, the importance of gut microbiota in ASD is supported by lower microbiota diversity in ASD patients [121]. Our analysis included six studies utilizing both targeted (probiotics) and broad-spectrum (FMT) intervention strategies. FMT-based strategies proved effective in reducing key ASD symptoms and inducing extensive metabolomic changes [70, 71]. However, a number of limitations, such as safety concerns for different populations, unclear duration of positive changes, and difficult donor selection limit the clinical application [122]. Despite these limitations and differences in protocol, FMT demonstrated more consistent improvements in social behavior compared to targeted interventions. Furthermore, recent methodological improvements in FMT delivery, particularly the development of encapsulated formulations, have reduced procedural complexity and enhanced standardization and repeatability [123].

Targeted microbiota interventions have a more predictable safety profile and higher translational significance [124]. However using individual bacterial strains produced modest [67] or limited benefits to a single behavioral trait [68]. Selected studies focused on classical probiotics (*Lactobacillus* and *Bifidobacteria spp.)* and future studies utilizing next-generation probiotics with a higher potential to synthesize biologically relevant metabolites could have better treatment outcomes [125]. Combining prebiotics and probiotics to achieve a synergistic synbiotic effect appears to be more effective strategy. Administration of *Lactobacillus reuteri C501* with inulin improved sociability, social novelty, and reduced repetitive self-grooming [69], indicating a more effective synergistic effect. Furthermore, specific bacterial and prebiotic interventions are generally well tolerated, easily standardized and repeatable, and present a lower practical and ethical barrier compared to FMT. Overall, microbiota-based interventions show promise in alleviating behavioral deficits associated with ASD though further mechanistic studies are required to establish molecular pathways underlying these beneficial effects.

The effectiveness of microbiota-based interventions highlights the importance of the host’s metabolic profile on the CNS. The consistent improvement in sociability and repetitive behaviors following metformin and pioglitazone treatment supports the effectiveness of metabolic modulators to treat both metabolic and behavioral symptoms of ASD. These effects are likely achieved through a reduction of oxidative stress, which is elevated in ASD patients [126]. Both metformin and pioglitazone were able to downregulate pro-inflammatory mediators in multiple brain regions, indicating a global anti-inflammatory effect. Additionally, both treatments had a positive effect on glutathione levels, suggesting regulation of redox activity. Overall, it can be theorized that alleviation of behavioral deficits was induced by a broad oxidative neuroprotective effect rather than modulation of neuronal activity or a single neurotransmitter pathway.

Metformin and pioglitazone exerted their beneficial effects without adverse metabolic effects, indicated by no changes in body weight during or after the treatment. This is particularly important since most of the antipsychotic medications used in ASD patients carry a substantial metabolic burden with well-known weight gain effects [127]. Naturally, the combination of metabolic agents and antipsychotic drugs becomes a potential strategy for reducing the metabolic side effects of current treatments. The synergistic effect of combined metformin and risperidone therapy in reducing levels of proinflammatory cytokines more effectively than either of these drugs alone, supports this theory [75]. These findings bring importance to systemic pathophysiology of ASD and the potential for metabolic drugs in a clinical setting.

Cannabinoid-based treatments represent another emerging strategy that targets the neuromodulatory and inflammatory components of ASD pathophysiology. The ECS regulates social behavior, synaptic plasticity, and neurodevelopment in general [128]. Current preclinical studies indicate that both phytocannabinoids (CBD, CBDV, THC) and complex cannabinoid mixtures (Avidekel, Erez oils, JZP541) can modulate ASD-like behaviors. However, these effects appear to be highly dose-dependent and cannabinoid-specific. Higher doses are reported to lower activity levels. The behavioral outcomes are caused by extensive transcriptional and neuroimmune changes that suggest a broad modulatory role on neuronal excitability and neuroinflammation. These transcriptional changes are especially prominent in the PFC and cerebellum with hundreds of affected genes. Most of these changes are connected to synaptic signaling and neural excitability, which are important molecular mechanisms affected in ASD. While promising, these findings do not address challenges in translating cannabinoid therapies to clinical practice, including regulatory and societal constraints, highlighting the need for changes in policy and public perception.

Analysis of selected studies suggests that interventions targeting the purinergic system, inflammation, and vitamin metabolism can yield measurable behavioral benefits in models of ASD. The common denominator of these approaches is the goal of restoring balance between metabolic, immunological, and neuroplastic processes. These results support the increasingly well-established systems approach, which views ASD as a disorder encompassing interdependent biological networks, ranging from mitochondria to the immune system to the gut-brain axis [129, 130]. Antipurinergic interventions based on suramin administration appear to act by inhibiting the excessive signaling of ATP and related nucleotides released from stressed cells. This finding is consistent with the ”cell danger response” and ”hyperpurinergy” hypotheses as possible pathogenic mechanisms of ASD [129]. Experimental studies report that suramin treatment improves social behavior while modulating mitochondrial gene expression and P2-receptor activity, suggesting coordinated regulation between purinergic metabolism and mitochondrial function. In contrast, anti-inflammatory approaches have produced the most consistent outcomes, including reductions in pro-inflammatory cytokines (IL-1β and TNF-α), elevations in anti-inflammatory mediators (IL-10 and LXA4), and structural restoration within the hippocampus and cerebellum, particularly increased neuronal density and Purkinje cell number. These neurobiological improvements align closely with enhanced social and cognitive performance, underscoring neuroinflammation as a promising therapeutic target [93, 96, 97]. Effective therapeutic paradigms appear to converge on modulation of the mitochondrial function, purinergic signaling, and neuroinflammation, often supplemented by nutritional regulators such as vitamin D₃ and retinoids. This integrative perspective supports the notion that persistent disruption of metabolic, immune, and nutritional signaling may sustain the ASD phenotype. It is also worth considering the roles of the gut-brain axis and the microbiota-immune-brain axis in modifying inflammation, gut barrier integrity, and neuroimmune signaling [131]. Dysbiosis and increased intestinal permeability (“leaky gut”) may amplify neuroinflammatory state of the brain, further justifying the need for research into interventions combining metabolic and microbiota-based therapies.

The current evidence base supports the idea that multi-pronged therapies targeting cellular metabolism, inflammation, and vitamin metabolism simultaneously have the greatest potential for translating into effective treatments for ASD. However, multiple limitations in preclinical studies reduce the significance of current findings. Less than 6% of the evaluated studies included male and female subjects. The absence of a standardized approach to examining both sexes in parallel limits the understanding of sex-related differences underlying the varying prevalence of ASD in boys and girls, as well as the potential for sex-dependent individualization of therapy within the development of personalized medicine. Another limitation is that many studies included in this review rely on single-dose designs; incorporating multiple-dose regimens and dose–response analyses could reduce potential misinterpretations and improve the robustness and interpretability of the findings, as study design critically influences effect estimation and translational validity in animal research [132]. An important point to emphasize here, in the context of current preclinical research on ASD, is that many behavioral tests assess domains that are relevant but not specific to autism alone. Commonly used measures, such as deficits in social interaction or repetitive behaviors, are present in many neuropsychiatric disorders, which limits the construct and face validity of the models used [102, 133]. This lack of specificity complicates the interpretation of treatment effects and underscores the need to develop more refined, translation-oriented behavioral paradigms. Additionally, a lack of standardization was observed in selecting and applying behavioral tests to assess ASD-like symptoms, which limits the ability to properly address key ASD behavioral deficits. Addressing these challenges would improve scientific rigor and increase the translational significance of interventional studies. It should also be emphasized that the role of the gut microbiota in ASD remains the subject of lively debate, and recent studies point to significant conceptual and methodological limitations, including issues related to causality, reproducibility of results, and confounding variables, which should also be kept in mind when interpreting the existing research findings, particularly with respect to causal inference and reproducibility [134]. Addressing these challenges will likely require improved stratification of ASD models, increased use of cross-model validation, and a stronger focus on mechanistically informed and translationally relevant endpoints [^134^]. While the presented preclinical findings constitute a significant contribution to the search for novel ASD therapies, it is crucial to exercise caution against premature clinical extrapolations that might detract from established evidence-based interventions, including behavioral and educational support [3, 135].

## Future directions

The results presented in this study suggest that targeting fundamental biological mechanisms holds promise for the future development of ASD therapies; however, their direct application in clinical practice remains limited at this time. The modest efficacy observed in human studies, such as in the case of oxytocin, likely reflects the significant heterogeneity of autism, differences in developmental trajectories, and the limited validity of existing preclinical models [136]. Therefore, although these approaches may provide a basis for hypothesis-driven interventions, their translation into effective treatments will require validation in large, statistically powerful, and rigorously controlled clinical trials.

Future research should investigate the current translational and methodological challenges highlighted in this review by adopting more standardized and integrative approaches. More studies should focus the preclinical study design on the clinical application. This could be achieved through the inclusion of both sexes, age-specific interventions, and the use of multiple ASD models that reflect the disorder’s genetic and environmental heterogeneity. Expanding behavioral analysis beyond sociability and repetitive behaviors to include anxiety, cognition, and activity could enhance the understanding of the full spectrum of behaviors. Mechanistic studies should increasingly combine molecular, transcriptomic, and metabolomic profiling to identify convergent pathways linking pharmacological, metabolic, and microbiota-targeted interventions. From the intervention side, the development of next-generation therapeutics such as stable oxytocin analogues, selective cannabinoid formulations, or synbiotic formulations would enable precision treatment.

The latest findings derived from large-scale longitudinal clinical data highlight the importance of distinguishing between two forms of autism exhibiting different developmental trajectories and polygenic architectures depending on the age at diagnosis [137]. Children diagnosed in early childhood (before age seven) are more likely to exhibit social and behavioral challenges during infancy and early childhood. At the same time, those identified later often showed typical early development but developed more subtle cognitive and behavioral difficulties over time. They were also more likely to have comorbid conditions, including ADHD and depression [137]. Consequently, a new challenge is to identify or develop preclinical models of both forms of ASD and to refine behavioral research paradigms to capture disorder-specific profiles more accurately when evaluating the efficacy of novel therapeutic interventions. This approach is essential for enhancing the translational relevance of preclinical research.

Given the rapid development of tools based on artificial intelligence and machine learning algorithms, it is essential to harness their potential for advanced analyses and integration of large-scale multi-omic datasets to identify novel early biomarkers of ASD, define molecular targets for new therapeutics, and develop libraries of new compounds with the potential to alleviate behavioral disturbances characteristic of individuals with ASD [137, 138]. Finally, longitudinal and cross-disciplinary studies integrating neuroscience, immunology, and microbiome research will be essential to translate preclinical insights into clinically effective, personalized therapeutic strategies for ASD.

## Supplementary information

Supplementary material 1

Supplementary material 2

Supplementary material 3

## Data Availability

The authors declare that all the data supporting the findings of this study are contained within the manuscript and Supplemental Materials.
