# Supplementary material for: Efficacy of pharmacological and microbiota-based therapies in preclinical models of autism spectrum disorder: a systematic review
Source: Mol Psychiatry. 2026 Jun 4;31(10):5823–61. doi: 10.1038/s41380-026-03663-8 (PMC13569414; doi:10.1038/s41380-026-03663-8)
Supplement: Supplementary file 1 [file 41380_2026_3663_MOESM1_ESM.docx]

Systematic Review Protocol

“Efficacy of Pharmacological and Microbiota-Based Therapies in Preclinical Models of Autism Spectrum Disorder: A Systematic Review”

# General Objective

To synthesize and critically evaluate preclinical evidence on the efficacy of pharmacological agents in ameliorating core behavioral phenotypes, neurotransmitter and hormonal changes observed in autism spectrum disorder (ASD) rodent models.

# Step 1: Define the Research Question

**PICO structure:**

**Population (P)**: Original articles investigating treatment of rodent models of ASD exhibiting core behavioral phenotypes.

1. **Inclusion Criteria**:

- Species: Mice and rats.
- Rodent models of ASD exhibiting core behavioral phenotypes, including:
  - Genetic models (SHANK3, Fmr1, etc.).
  - Environmental induction models (valproic acid exposure, maternal immune system activation).
  - Idiopathic: inbred strains with ASD-like behaviors.
- Age: All stages of development of postnatal development.
- Sex: Both male and female animals.

2. **Exclusion Criteria**:

- Non-rodent models.
- Human studies.
- Rodent models not specifically validated with ASD-like phenotype (anxiety, depression, etc.) without ASD-specific behaviors).

**Intervention (I)**: Pharmacological and microbiome-targeted interventions:

1. Pharmacological agents:

- Neurotransmitter modulators
- Immunomodulators
- Hormonal therapy

2. Microbiome-targeted interventions:

- Antibiotics
- Fecal microbiota transplantation (FMT)
- Prebiotics, probiotics, postbiotics and their combinations (synbiotics).

**Comparison (C)**:

1. Control groups who receive vehicle, sham procedure or no treatment.
2. Baseline of experimental group

**Outcome (O)**:

1. Primary outcomes, ASD-like behaviors frequently observed in ASD rodent models:

- Changes in social interactions
- Alterations in repetitive, stereotypical behaviors
- Changes in anxiety and activity levels

2. Secondary outcomes:

- Neurotransmitter changes
- Hormonal changes
- Alterations in inflammatory markers
- Gut microbiota diversity changes
- Metabolite changes

**Formulate the Research Question**

What is the efficacy of pharmacological and microbiota-based interventions in improving core behavioral phenotypes, neurotransmitter profiles, and hormonal alterations in rodent models of ASD?

**Justification**

ASD is a complex neurodevelopmental condition that affects how an individual perceives and engages with environment and others, characterized by deficits in social communication and repetitive behaviors. ASD exhibits has high prevalence, with current estimates at approximately 1 in 100 individuals. Current interventions have minimal success at ameliorating core behavior symptoms of ASD. Pharmacological treatment strategies targeting core ASD symptoms remain limited, and current therapeutic options have limited efficacy with uncertain safety profiles.

Preclinical rodent models of ASD have been instrumental in starting to understand the complex etiology and mechanisms underlying the development of the disorder and in testing potential therapeutic strategies. While a growing number of animal studies suggest beneficial effects from novel pharmacological and gut-microbiota based interventions on ASD-like behaviors and related neurochemical alterations, the data remains heterogeneous, evidence fragmented, and largely unevaluated in a systematic manner. A systematic review in this field could help to identify the most promising therapeutic strategies for clinical translation, highlight existing evidence, prevent unnecessary replication and use of animals, reveal gaps in methodology and biases in the reporting.

# Step 2: Comprehensive Literature Search

**Database Selection**

A systematic literature search will be conducted using PubMed, Web of Science, ScienceDirect databases. These databases were chosen due to their extensive coverage of biomedical literature.

All retrieved records will be managed using “Microsoft Excel” and “Zotero” to facilitate deduplication, screening, and selection.

**Inclusion and Exclusion Criteria**

1. **Inclusion Criteria:**
   - Original preclinical studies published in peer-reviewed journals within the past 15 years.
   - Studies assessing the effect of pharmacological or microbiome-targeted interventions.

- Studies reporting ≥1 ASD-associated behavior.
- Studies utilizing pharmacological or non-pharmacological interventions.
- Study design: *In vivo* experimental studies.
- Studies must specify intervention dose, duration, and administration route.
- Studies published in English.

2. **Exclusion Criteria:**

- Reviews, meta-analyses, commentaries, editorials, conference abstracts, case reports without original data.
- *In vitro, ex vivo,* or computational modeling studies.
- Reports with incomplete data on intervention protocols, outcome measures, or statistical analysis.
- Prenatal intervention studies.
- Studies reporting only qualitative data or behavioral observations.
- Publications utilizing duplicating datasets.
- Small sample sizes (n < 4 per group) unless justified by power analysis.
- Neonatal-only studies without postnatal behavioral or neurochemical outcomes.
- Articles not available in full text.
- Non-English publications

**Constructing Search Strategy**

A comprehensive search strategy will be utilized to identify relevant preclinical studies assessing pharmacological and microbiome-targeted interventions in rodent models of ASD.

1. **Identifying Key Concepts**

Key concepts derived from the research question the PICO framework are:

- Autism Spectrum Disorder
- Rodent models
- Pharmacological interventions
- Microbiome-based interventions

2. **Keywords and Combinations:**

- **Primary Keywords:**
  - “Autism Spectrum Disorder”
  - “ASD animal model”
  - “mice”
  - “rats”
  - “intervention”
  - “treatment”
  - “microbiome”
  - “microbiota”
  - “supplementation”
  - “administration”
- **Combinations:**
  - “Autism Spectrum Disorder AND intervention AND mice”
  - “Autism Spectrum Disorder AND intervention AND rats”
  - “ASD animal model AND intervention”
  - “ASD animal model AND treatment”
  - “ASD animal model AND supplementation”
  - “ASD animal model AND microbiota”
  - “ASD animal model AND microbiome”

3. **Filters:**

Appropriate filters will be applied to limit the search according to the including and exclusion criteria:

- Publication date filter
- Study type filter
- Species filter
- Language filter

**Documenting the Strategy**

To ensure transparency, reproducibility, and compliance with PRISMA guidelines, the search strategy will be documented as follows:

- Full search string, name and platform of the database, filters used, date the search was performed and number of publications retrieved.

A PRISMA flow diagram will be used to transparently report the study selection process, including:

- Total number of records identified from all sources.
- Number of duplicates removed.
- Number of studies screened, excluded, assessed for full text, and included.

This diagram will be included in both the protocol and the final review manuscript.

# Step 3: Study Selection

**Initial Screening**

All articles retrieved from the databases will be deduplicated by automated and manual means. After the deduplication, two reviewers will independently analyze titles and abstracts of all studies according to our inclusion and exclusion criteria. Discrepancies between the two reviewers will be resolved with a consultation from a third reviewer.

**Full Text Review**

Articles that passed initial screening will undergo a full-text eligibility assessment by three reviewers. Each reviewer will independently assess whether each publication meets all inclusion criteria. If an article is excluded at this stage, the specific reason for exclusion will be recorded.

# Step 4: Quality Assessment of Studies

To ensure methodological rigor and minimize bias, the quality of included preclinical studies will be assessed using validated tools.

**Quality Assessment Tools**

1. The SYRCLE’s Risk of Bias (RoB) tool for animal studies will be used to assess the internal validity of each study. This tool was developed using the Cochrane RoB tool and is specifically designed to improve critical appraisal of evidence from animal studies. Available at:

<https://bmcmedresmethodol.biomedcentral.com/articles/10.1186/1471-2288-14-43>

**Quality Criteria**

1. The SYRCLE’s Risk of Bias evaluates studies in domains:

- Sequence generation
- Baseline characteristics
- Allocation concealment
- Random housing
- Blinding (during experiment and outcome assessment)
- Incomplete outcome data
- Selective outcome reporting
- Other biases (e.g., conflicts of interest)

**Documenting the Assessment**

The quality assessment process will be thoroughly documented. Each study will be assessed independently by two reviewers using the SYRCLE checklist. Scores for each domain will be recorded in a table and a “traffic light” plot for better visualization.

# Step 5: Data Extraction

A standardized data extraction form will be used to capture study characteristics, methodological details, intervention specifics, outcome measures, and main findings.

**Data Extraction Sheet**

A customized data extraction form will be developed in Excel software. The following will be collected:

- Study identification with general information (author, year)
- Animal model type and characteristics (species, strain, ASD model, sex, age)
- Intervention (type, name of compound, dosage, route, frequency, duration)
- Outcome measures and findings (behavior assessment, gene expression, neurotransmitter or hormonal measures, gut microbiota changes)

**Double Review**

Two reviewers will independently extract data to ensure accuracy and minimize bias. Any discrepancies in extracted data will be arbitrated by a third reviewer.

# Step 6: Analysis and Synthesis of Results

**Narrative synthesis**

Studies will be organized into thematic categories described in a narrative synthesis. This synthesis will explore patterns, consistencies, and discrepancies in the data, while highlighting factors that may explain found differences and data heterogeneity. Results will be grouped and discussed thematically and presented in summary tables. Key factors that will be addressed in narrative synthesis:

- Type of intervention (main outcomes per intervention type, number of studies contributing to each outcome)
- Direction, strength, and significance of findings
- Study quality and risk of bias (confidence in the evidence base)
- Differences in models

**Documenting Findings**

All assessed data will be documented in three main ways:

- PRISMA Flow diagram
- Summary tables, grouping results by intervention type and outcome domain
- Visual representation

# Step 7: Writing the Manuscript

1. **Introduction:** Context, background information, justification, the research question.
2. **Methods:** Description of the search strategy, study selection, eligibility criteria, data extraction and management, PICO model, SYRCLE methodology, data synthesis.
3. **Results:** PRISMA flow diagram, results tables, visual representation.
4. **Discussion:** Interpretation and contextualization of findings, strengths and limitations,
5. and implications for future directions, translation to clinical relevance.
6. **Conclusion:** The main concise statement about the overall conclusions, practical implications, future directions.
